# Reinforcement learning for policymaking in epidemic control: A scoping review

**DOI:** 10.1371/journal.pone.0351176

**Published:** 2026-06-08

**Authors:** Oleksandr Bolshov, Dmytro Chumachenko

**Affiliations:** 1 Department of Computer Science and Information Technology, ‌‌National Aerospace University “Kharkiv Aviation Institute”, Kharkiv, Ukraine; 2 Mathematical Modelling and Artificial Intelligence Department,‌‌ National Aerospace University “Kharkiv Aviation Institute”, Kharkiv, Ukraine; Nanyang Technological University, SINGAPORE

## Abstract

**Background:**

Managing an epidemic demands policies that respond at the pace of the outbreak. Conventional rule‑based interventions struggle to keep up, prompting interest in reinforcement learning (RL) for designing non‑pharmaceutical interventions (NPIs). However, current evidence is fragmented across diverse models and reporting styles.

**Objectives:**

To systematically map how RL is applied for epidemic NPI design, describe modeling choices, algorithm architectures, evaluation practices, and identify trends and research gaps.

**Methods:**

Peer-reviewed studies (2014–2025, English) that applied deep RL to select NPIs were retrieved from IEEE Xplore, ACM Digital Library, ScienceDirect, and Scopus, searched on December 23, 2025. Reference list scanning supplemented database results. Predefined data items (bibliographic details, epidemic and RL model characteristics, experiments, validation methods, outcomes) were charted and summarized descriptively.

**Results:**

Of 512 retrieved records, 10 met the inclusion criteria, and three additional studies were identified via reference-list scanning, yielding 13. Five employed value‑based methods, four policy‑gradient, and four hybrid; one study additionally incorporated model-based planning. Six simulations relied on compartmental models, six on agent‑based models, and one on a hybrid model. Action spaces were predominantly discrete restriction levels. Five studies incorporated sequence-modeling techniques to include temporal context into a state space. Eleven studies designed reward functions as a trade-off between pandemic severity and socio-economic cost. According to the reviewed studies, RL policies across various settings outperform heuristic, rule-based, and historical baselines in reducing infections, deaths, or lockdown duration while limiting economic loss.

**Conclusions:**

RL shows promise for adaptive epidemic control. Comparison is hampered by simplified economic costs, inconsistent calibration rigor, varied evaluation metrics, and limited uncertainty or policy robustness analysis. Future work should establish common benchmark environments and reporting standards, incorporate empirically grounded economic and behavioral models, adopt uncertainty-aware and probabilistic RL, develop more sophisticated control spaces, investigate more advanced algorithms, and validate learned policies prospectively to enable real-world deployment.

## Introduction

### Rationale

Epidemic outbreaks present complex decision-making challenges that require adaptive interventions [1]. Traditional modeling and control strategies, such as rule-based systems or fixed policy heuristics, often struggle to capture the dynamics of evolving epidemic environments or to optimize long-term outcomes under uncertainty [2]. Reinforcement learning (RL) mitigates this limitation by iteratively refining intervention strategies within simulated environments, where real‑world experimentation would be impractical or unethical [3,4].

Interest in RL intensified during the COVID‑19 pandemic, when researchers used it to design non‑pharmaceutical interventions such as mask mandates, mobility restrictions, social distancing measures, etc. This approach has demonstrated that RL can balance competing objectives such as infection control and economic preservation. Agent-based modeling (ABM) further enhances this potential by simulating the behavior and interactions of heterogeneous individuals, yielding more realistic and granular simulations of epidemic dynamics and policy effects [5].

Nevertheless, the field remains fragmented. Studies differ in algorithmic choices, modeling assumptions, and evaluation metrics, complicating cross‑study comparison. A comprehensive mapping of how RL methods are currently employed for epidemic policymaking is lacking. This scoping review addresses this gap by analyzing the state of research at the intersection of epidemic control and RL.

### Objectives

This review aims to map the current research landscape on the use of RL methods for epidemic control policymaking. Specifically, the objectives are:

1. To identify and categorize studies that apply RL algorithms to NPI design in epidemic settings.
2. To analyze how these studies model epidemic dynamics, agent behaviors, policy environments, and what model validation techniques are used.
3. To characterize the RL methods employed, including algorithmic types, reward design, and state and action spaces.
4. To identify methodological patterns, research gaps, and potential directions for future work in this domain.

## Methods

This scoping review followed the PRISMA extension guidelines for scoping reviews (PRISMA-ScR) [6]. No review protocol was registered for this study.

### Eligibility criteria

#### Language and publication type.

We included only studies published in English. Only original research articles from peer-reviewed journals were considered. We excluded studies such as conference abstracts, editorials, reviews, opinions, or other non-peer-reviewed materials,

#### Time frame.

The review focused on studies published between 2014 and 2025. This time frame begins with the resurgence of deep learning as a transformative tool in artificial intelligence and continues through the COVID-19 pandemic. The pandemic marked a period of unprecedented growth in healthcare research, including the application of machine learning to manage public health crises. We excluded older studies, as they are less likely to reflect the modern AI/ML techniques relevant to our objectives.

### Study design.

We included only studies that employ deep RL techniques and, preferably but not necessarily, ABM. We excluded studies that rely exclusively on statistical modeling, rule-based decision-making, or other non-RL methods.

#### Application context.

This review included only studies addressing epidemic control through policymaking, where a RL agent acts as a decision-maker responsible for implementing non-pharmaceutical interventions. Eligible studies modeled agents selecting among intervention strategies, such as lockdowns, social distancing, or mask mandates, to mitigate disease spread.

We excluded studies if they did not align with this policymaking application. In particular, we did not consider works focused on crowd avoidance, resource allocation, or general applications of artificial intelligence outside the context of epidemic control. Studies addressing epidemic-related problems without active policy decision-making, such as forecasting, contact tracing, or descriptive modeling without an intervention agent, were also excluded.

The decision to focus exclusively on NPI policymaking and exclude other types of applications is dictated by the fact that all of these applications have fundamentally different objects of influence, objective functions, and methodologies. NPIs aim to alter system dynamics by reducing transmission through social regulation. In contrast, resource allocation, for example, is primarily a logistical optimization problem. Including all of these applications would have blurred the focus of this work. By narrowing the application context to NPIs, this review aims to provide a more rigorous comparative analysis of RL architectures specifically tailored for the population-level social interventions and socio-economic trade-offs.

### Search strategy

The search was conducted on December 23, 2025, in computer science and multidisciplinary databases, specifically IEEE Xplore, ACM Digital Library, ScienceDirect, and Scopus.

Three main categories were identified, each with corresponding keywords:

1. Artificial Intelligence: reinforcement learning.
2. Agent-Based Modeling: multi-agent systems, agent modeling, agent-based modeling, intelligent agents, autonomous systems.
3. Epidemics: epidemics, pandemics, outbreak, infectious disease, public health.

For Scopus, IEEE Xplore, and ACM Digital Library, the search string was the following:

“reinforcement learning” AND (“multi-agent systems” OR “agent modeling” OR “agent-based modeling” OR “intelligent agents” OR “autonomous systems”) AND (epidemics OR pandemics OR outbreak OR “infectious disease” OR “public health”).

For Science Direct, due to search string length restrictions, it was shortened to the following:

“reinforcement learning” AND (“multi-agent systems” OR “agent modeling” OR “agent-based modeling”) AND (epidemics OR pandemics OR “infectious disease” OR “public health”).

Keywords were searched in the Title, Abstract (Summary), and Keywords fields.

### Study selection

The selection process was conducted using the Covidence information system. Both authors independently screened the titles and abstracts of eligible studies, followed by a full-text review of those that passed the initial screening. Any discrepancies were resolved through discussion.

### Data extraction

The following key components were extracted from all included studies: bibliographic information, methods, experiments, results, and discussion. Data items for each component were defined as follows:

1. Bibliographic details: title, authors, publication year, and journal.
2. Methods: reinforcement learning algorithm, epidemic modeling approach, and software for simulation.
3. Experiments: RL-specific characteristics, experimental design, and model validation techniques.
4. Results: epidemic mitigation outcomes and RL-related findings.
5. Discussion: stated limitations and suggested future research directions.

Data extraction was conducted by OB and independently verified by DC. Disagreements were resolved through discussion.

### Synthesis of results

Comparative tables were created to summarize study characteristics, including epidemic models, RL algorithms, intervention types, etc. The synthesis is focused on identifying common trends, differences in RL methodologies, and potential research gaps. Findings were analyzed qualitatively and quantitatively, highlighting strengths and limitations across studies.

### Use of artificial Intelligence tools

We used ChatGPT (model o3) exclusively to improve the readability and stylistic clarity of this manuscript. All AI-generated suggestions were carefully reviewed, revised, and validated by the authors to ensure factual accuracy. The final wording, scientific interpretations, conclusions, and limitations reflect the authors’ own ideas and intellectual contributions. The authors bear full responsibility for the integrity and originality of the content presented.

## Results

### Study selection

**Fig 1:**
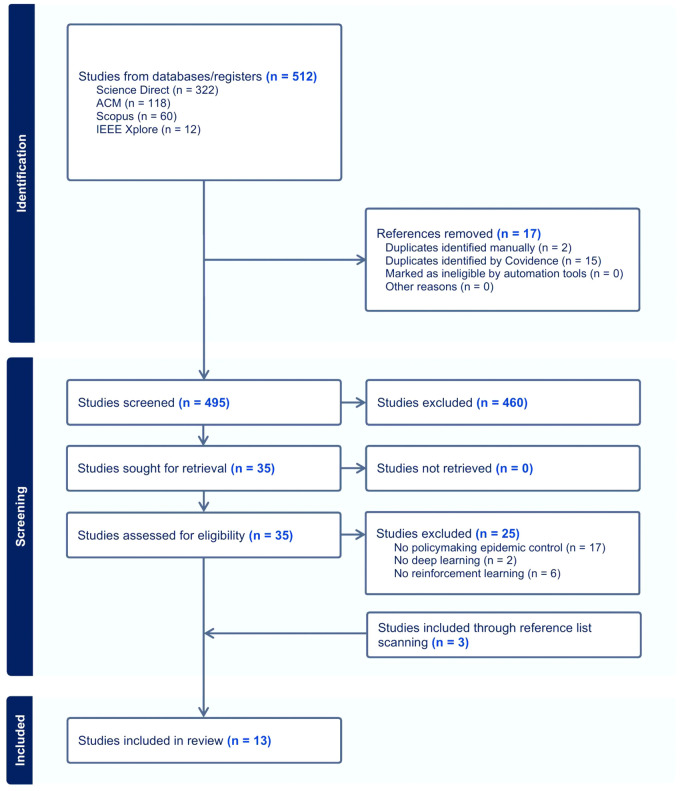
PRISMA flow diagram.

Fig 1 presents the PRISMA flow diagram. The keyword search identified 512 studies, with 17 duplicates removed. During the screening phase, 460 papers were excluded as irrelevant. Of the 35 papers assessed in the full-text review, 25 were excluded for the following reasons: no policymaking epidemic control (*n* = 17), no deep learning (*n* = 2), and no RL (*n* = 6). Additionally, three studies were included through reference list scanning.

### Study characteristics

Table 1 contains key characteristics of the selected studies, including bibliographic details (title and authors), the RL algorithm, and the epidemic dynamics model.

**Table 1:** General characteristics of the studies.

#	Authors	Title	RL algorithm	Epidemic dynamics
1	Shuvo et al [7]	Advancing pandemic preparedness through a data-driven hybrid simulation model	Value-based	Hybrid: SEIHRD with ABM-simulated interactions
2	Zhang et al [8]	A data-driven pandemic simulator with reinforcement learning	Value-based	Agent-based
3	Libin et al [9]	Deep reinforcement learning for large-scale epidemic control	Policy-based	Compartmental: Age-structured stochastic SEIR
4	Zong and Luo [10]	Reinforcement learning based framework for COVID-19 resource allocation	Policy-based	Agent-based
5	Guo et al [11]	PaCAR: COVID-19 pandemic control decision making via large-scale agent-based modeling and deep reinforcement learning	Value-based	Agent-based: Simulates city-scale population heterogeneity
6	Hu et al [12]	Decentralized graph-based multi-agent reinforcement learning using reward machines	Hybrid	Compartmental: Network of 20 regional SIQHRD models
7	Du et al [13]	HRL4EC: Hierarchical reinforcement learning for multi-mode epidemic control	Policy-based	Compartmental: Multilateral-Impact-Driven SEIR
8	Kompella et al [14]	Reinforcement learning for optimization of COVID-19 mitigation policies	Hybrid	Agent-based
9	Wan et al [15]	Multi-objective model-based reinforcement learning for infectious disease control	Value-based^1^	Compartmental: Generalized SIR
10	Ohi et al [16]	Exploring optimal control of epidemic spread using reinforcement learning	Value-based	Agent-based: Grid-based individual movement
11	Luo et al [17]	Architecting urban epidemic defense: A hierarchical region-individual control framework for optimizing large-scale individual mobility interventions	Policy-based	Agent-based: Stochastic SLIR dynamics with ABM-simulated daily activities
12	Samadi et al [18]	Deep Reinforcement Learning for Epidemic Control of a Networked SIS Model	Hybrid	Compartmental: Networked SIS dynamics
13	Luo et al [19]	H2-MARL: Multi-agent reinforcement learning for Pareto optimality in hospital capacity strain and human mobility during epidemic	Hybrid	Compartmental: Dynamic-SIHR metapopulation model

^1^The study employs a model-based planning framework and also incorporates a tabular policy search variant alongside the deep learning value-based approach.

#### Reinforcement learning algorithms.

RL algorithms can be broadly classified into model-based and model-free approaches [20]. Model-based RL involves training an agent on a learned or predefined model of the environment, allowing it to simulate future states before making decisions. While this approach can be sample-efficient, it is computationally expensive and requires an accurate environment model, which is often unavailable or impractical for complex real-world problems. Nevertheless, study [15] demonstrates that an accurate model for the epidemic control problem can be constructed. Their framework leverages a Bayesian-updated generalized SIR model to simulate future epidemic trajectories via Monte Carlo rollouts, which allows an agent to predict future outcomes to plan its interventions.

In contrast, model-free RL does not rely on an explicit model of the environment. Instead, agents learn optimal policies through interaction, making decisions based on past experiences. Due to its flexibility and ease of implementation, model-free RL is a more popular approach, and all of the selected studies in this review employ model-free methods.

Model-free RL algorithms can be further categorized into value-based, policy-based, and hybrid approaches:

1. Value-Based methods. These methods optimize a value function, which estimates the long-term expected reward for each state-action pair. The agent indirectly derives its policy by selecting the action with the highest estimated value. Deep Q-Network (DQN) and its variants (Double DQN, N-step DQRL, etc.) fall into this category. These methods suit discrete action spaces but struggle with high-dimensional continuous control tasks.
2. Policy-Based methods. These methods learn the policy directly by parameterizing and optimizing it using gradient-based techniques. Unlike value-based methods, they are better suited for problems with continuous or stochastic action spaces. Examples include Proximal Policy Optimization (PPO) and Actor-Critic methods.
3. Hybrid approaches. These methods combine value function approximation and policy optimization, leveraging the strengths of both paradigms. For example, Soft Actor-Critic (SAC) uses actor-critic architectures where a value function (critic) stabilizes policy learning while optimizing a direct policy (actor).

Among the reviewed studies, value-based methods are used in studies [7,8,11,15,16], primarily relying on DQN and its variants. Policy-based RL is applied in [9,10,13,17]. Hybrid approaches are employed in [12,14,18,19].

#### Epidemic dynamics modeling.

The reviewed studies utilize two primary epidemic modeling approaches: compartmental models and agent-based models. While these are often considered distinct methodologies, some studies integrate both into hybrid models, combining the strengths of each approach.

Compartmental models divide the population into distinct health states and describe transitions between these states using differential equations, assuming a homogeneous population [21,22]. These models are particularly useful for large-scale epidemic forecasting and intervention planning, as they estimate infection trends, hospitalization needs, and intervention effects at the population level [23]. Among the reviewed studies, variations of the SIR (Susceptible-Infected-Removed) and SEIR (Susceptible-Exposed-Infected-Removed) models were commonly used. Study [9] followed the standard SEIR formulation, while others expanded it with additional compartments. Study [7] employed the SEIHRD model, introducing a hospitalization state and splitting the “removed” compartment into “recovered” and “deceased.” Study [12] took a slightly different approach, using an SIQHRD model, where Q represents a quarantined state. Study [13] modified the standard SEIR model into MID-SEIR, incorporating external intervention effects. Study [15] proposed a stochastic generalized SIR (GSIR) model in which intervention levels directly affect the infection rate. Study [18] utilized a networked SIS model to represent infection dynamics without permanent immunity. Study [19] expanded the SIR framework with a Dynamic SIHR model, including a hospitalization state and online parameter updates.

Unlike compartment models, ABM captures population heterogeneity, simulating epidemic spread at the individual level, where agents represent people with unique characteristics, behaviors, and movement patterns. Transmission occurs through direct agent interactions, making ABM useful for modeling mobility, social behavior, and localized outbreaks [5]. Six studies, [8,10,11,14,16,17], used a purely agent-based approach. In these studies, both transmission and disease progression were modeled at the individual level, with infected agents transitioning through disease states based on probabilistic state changes, rather than differential equations applied to the population.

One study [7] combined ABM with a compartmental model, creating a hybrid model that balances realistic agent interactions with structured epidemic progression. In this case, ABM governed agent interactions and exposure, while the compartmental model dictated infection progression rate.

Table 2 summarizes the key RL components across studies, including algorithm type, state representation, action space, and reward formulation.

**Table 2:** RL characteristics of the studies.

#	Study	Algorithm	State space	Action space	Reward function
1	Shuvo et al [7]	N-step DQRL	Health-state variables, previous action	3 restriction levels + no action	Pandemic severity vs policy strictness
2	Zhang et al [8]	DQN	Health-state variables	6 composite restriction levels	Pandemic severity vs socio-economic costs
3	Libin et al [9]	PPO	Health-state variables for four age groups, the budget of school closures	School closures and openings	Minimization of new infections
4	Zong and Luo [10]	Multi-agent recurrent attention actor-critic + gated recurrent unit (MARAAC)	7-day history of epidemic transmission data, the agent’s corresponding rewards	5 composite restriction levels	Pandemic severity vs policy strictness
5	Guo et al [11]	DQN + Transformer	Daily and cumulative number of ascertained cases, previous actions, number of vaccinated people	4 composite restriction levels	Pandemic severity vs policy strictness
6	Hu et al [12]	Decentralized graph-based MARL with reward machines and actor-critic structure (DGRM)	Health-state variables, number of days in a severe situation, number of days adopting lockdown	4 movement restriction levels	Pandemic severity vs policy strictness
7	Du et al [13]	Hierarchical PPO	Health-state variables, degree of human contact	High-level: single- or multi-mode intervention. Low-level: intensity of interventions	Pandemic severity vs economic costs
8	Kompella et al [14]	SAC	Global infection summary for critic (only during training), global testing summary for actor, current restriction level	5 composite restriction levels	Pandemic severity vs policy strictness
9	Wan et al [15]	DQN^2^	Health-state variables	3 restriction levels	Pandemic severity vs economic costs
10	Ohi et al [16]	Double DQN + LSTM	Health-state variables, *R*_0_, economic state, current restriction level	3 movement restriction levels	Pandemic severity vs economic costs
11	Luo et al [17]	Multi-agent PPO + LSTM	Health-state variables, daily increase in infectious cases, distribution of mobility interventions, number of high-risk residents	3 intervention types with 5 intensity levels	Pandemic severity vs socio-economic costs
12	Samadi et al [18]	Multi-agent DDPG	Vector of node infection probabilities, weighted contact matrix of the local subgraph, current time-step	Continuous adjustment of contact intensities for all edges in the subgraph, constrained by a fixed budget	Reduction in the average infection probability
13	Luo et al [19]	Multi-agent reinforcement learning for Pareto optimality in hospital capacity strain and human mobility (H2-MARL)	Health state variables and their daily changes, accumulated restriction loss, current inter-regional mobility demand	Continuous adjustment of inter-regional mobility quotas	Hospital capacity strain vs socio-economic mobility loss

^2^The study employs a model-based planning framework and also incorporates a tabular policy search variant alongside the DQN approach.

#### Reward function.

Almost all studies design the reward function to reflect a trade-off between pandemic severity and socio-economic costs. In some studies, economic costs are modeled explicitly, using quantifiable proxies such as productivity loss or healthcare burden. In others, socio-economic costs are represented implicitly by penalizing the intensity or duration of interventions, such as lockdowns, which indirectly reflect the economic disruption. Study [8] also includes a psychological factor that reduces the reward when stricter restrictions are applied. Additionally, studies [11] and [14] penalize frequent changes in restrictions to encourage policy stability. Studies [9,18] take a different approach by focusing solely on epidemic suppression, incentivizing the agent to optimize epidemiological outcomes without economic considerations. This design encourages proactive intervention to slow infection spread rather than balancing competing societal costs.

Some studies propose more complex reward designs. Study [12] departs from the standard scalar reward design using a reward machine to encode a non‐Markovian reward structure. This approach decomposes the overall reward into high‐level stages based on epidemic severity and intervention measures. Study [19] introduces a dynamic weighting mechanism using the entropy weight method to automatically adjust the priorities between healthcare strain and mobility loss based on their real-time criticality.

#### Action space.

In studies [8,10,11,14,17], the agent selects among discrete restriction levels, ranging from minimal or no restrictions to full lockdown. In studies [8,10,11,14], these levels represent a composite of multiple interventions, such as social distancing, isolation, school and business closures, mask mandates, etc. In study [17], the agent independently adjusts the intensity levels for isolation, quarantine, and confinement Studies [7] and [15] adopt a similar abstraction but do not explicitly decompose restriction levels into individual measures; instead, these levels only influence the reward function and, in the case of [15], affect the transition model. In study [9], the agent controls only the school closures.

Studies [12,16,19] model movement restrictions. Study [12] presents four intervention types: no restrictions, forced social distancing, regional movement restrictions, or a combination of these measures. Similarly, study [16] offers three movement restriction levels: no restrictions, social distancing, and full lockdown. On the contrary, study [19] employs a continuous mobility quota matrix to regulate population flows between administrative regions.

Study [13] employs hierarchical RL, where at the high level, the agent selects either a single intervention or a combination of them, while at the low level, it determines the intensity of the selected restrictions. Study [18] utilizes a continuous action space to directly modify contact network edge weights, constrained by a fixed intervention budget.

#### State space.

The state spaces in the reviewed studies primarily consist of health-state variables, such as the number of cases in different population health states (susceptible, infectious, recovered, etc.), sometimes combined with past actions taken. Some studies incorporate additional elements. Namely, study [9] includes the remaining budget for school closures. Study [11] integrates daily and cumulative ascertained cases, prior interventions, and vaccination numbers. Study [12] utilizes a graph-based multi-agent system where each agent’s state also includes the number of days in severe conditions and lockdown history. Similarly, study [18] employs a graph-based state consisting of node-level infection probabilities and the current network adjacency matrix. Study [13] integrates human contact degrees to model social interactions. In study [19], the state space incorporates mobility demand and an accumulated restriction loss to represent the historical impact of past interventions. In study [14], the infection status of the population is only partially observable, with available data limited to testing results and hospitalizations. Study [17] includes regional infection increments, intervention distributions, and the count of individuals with high infection risk. In addition, studies [10–12,16,17] differ by employing sequence modeling techniques, allowing them to process historical epidemic trends.

#### Epidemiological validity.

The epidemiological validity of reinforcement learning policies depends on the accurate parameterization of disease transmission and progression models, ensuring that the model accurately reflects the underlying biological reality. Across the reviewed studies, researchers handle the basic reproductive number *R*_*0*_ using three primary approaches: as an explicit input, an emergent simulation property, or a dynamically estimated variable. Studies [9,11,13,17,18] represent the first approach and define explicit *R*_*0*_ values to characterize specific pathogens or variants. The second strategy, where *R*_*0*_ serves as an emergent property of individual interactions tuned to match the historical trends, is used in studies [8,10,14,16]. Finally, the third approach focuses on real-time estimation from empirical data [7,12,15,19].

Biological parameters defining disease progression, such as incubation periods, latent periods, and fatality ratios, are mostly based on clinical reports and peer-reviewed medical literature. Studies [7,8,10,14,17,19] use COVID-19 clinical investigations to justify disease progression parameters and health-state transitions. Study [15] relied on estimates from the SARS pandemic to set priors for disease features. Study [11] utilized variant-specific data from medical journals regarding vaccine effectiveness. Study [18] relies on assumed parameters for a synthetic networked SIS model to demonstrate algorithmic performance without specific clinical grounding. Studies [9,10,17] used data from national statistical, census, and demographic databases to tailor simulation environments to specific geographic contexts.

The justification of contact rates and social mixing patterns relies on either empirical behavioral data or structural assumptions. Study [9] integrated age-structured contact matrices from an internet-based social contact survey to capture social mixing patterns within a meta-population framework. Studies [17,19] utilize large-scale empirical mobility data from mobile phones to parameterize contact patterns through observed population movement. Studies [12,13,18] approximate interaction frequencies through administrative boundary analysis or synthetic network topologies, where transmission parameters are assumed rather than empirically sourced.

Researchers used various methods to make their models match real epidemics. Most studies used COVID-19 data from regions like China, Sweden, Canada, Italy, and the USA. The calibration rigor differs across studies: some studies relied on literature-based assumptions and manual alignment with historical peaks, while others used statistical methods to derive parameters from health surveillance data. Validation was primarily achieved by reproducing historical outbreak trajectories or comparing simulation outputs against compartmental models. Almost all authors conducted sensitivity analysis to see how changes in key epidemiological parameters affect policy performance or epidemic dynamics. Table 3 summarizes validation and calibration methods used in the reviewed studies.

**Table 3:** Calibration and validation methods.

#	Study	Data source	Calibration methods	Validation methods	Sensitivity analysis
1	Shuvo et al [7]	COVID-19 data from Canadian provinces (Ontario, Quebec, BC, Alberta)	Nonlinear Least Squares (NLS) for model parameters estimation	Out-of-sample testing comparing predicted vs. actual cases using RMSE and MAE	Impact of initial parameter variations on the stability of NLS estimation
2	Zhang et al [8]	COVID-19 data from Poland, China, and the US	Use of statistical reports to set incubation and fatality rates	Replication of historical active, confirmed, and death case trajectories	Univariate analysis of five NPI policy variables on peak active cases
3	Libin et al [9]	2009 H1N1 influenza data and census/mobility data from Great Britain	Patch model for force-of-infection parameter tuning	Replication of the two-peak curve of the 2009 H1N1 pandemic	Impact of varying *R*_*0*_ (1.8 vs. 2.4) on the proportion of the population infected
4	Zong and Luo [10]	COVID-19 data from Sweden and demographic data from the US	Bayesian optimization for infection spread and social distancing rates	Alignment with Swedish historical data for time-to-peak mortality	Impact of infection spread rates and scaling factors on peak infections and mortality
5	Guo et al [11]	COVID-19 data from King County (USA) and Beijing (China)	Agent-based model tuning to census data and real infection curves	Replication of Alpha and Delta variants trends and vaccination scenarios	Robustness testing under uncertainty with uniformly distributed *R*_*0*_
6	Hu et al [12]	COVID-19 data from the Italian Civil Protection Agency	Use of real health data to set initial model state variables	Alignment with a pre-validated pandemic model	Not reported
7	Du et al [13]	COVID-19 data from Changchun and Shanghai	Use of urban-specific attributes to adjust transmission risks	Comparison against official reports for cumulative infected cases	Impact of *R*_*0*_ variations (7.5–8.5) on outbreak intensity
8	Kompella et al [14]	COVID-19 data from Sweden	Grid search for deaths and critical cases to match historical “time-to-peak”	Replication of unrestricted pandemic dynamics in Sweden	Impact of spread rates, contact rates, and gathering sizes on mortality levels
9	Wan et al [15]	COVID-19 data from six Chinese cities and SARS-related priors	Online Bayesian framework for infection and removal rate estimation	Out-of-sample prediction and Leave-One-Out Cross-Validation	Impact of model structure (SIR vs. SEIR) and *R*_*0*_ shifts on robustness
10	Ohi et al [16]	COVID-19 data from China	Population density adjustments for target *R*_*0*_ alignment	Comparison with state trajectories of standard SEIR model	Impact of population density on contact probability and disease surge
11	Luo et al [17]	Demographic and mobility data from Shenzhen, contact patterns from Shanghai	Contact network calibration based on Shanghai contact patterns	Statistical comparison of synthetic population attributes against census and mobility surveys	Impact of *R*_*0*_ variations (2.0–6.5) and risk estimation windows on infection rates
12	Samadi et al [18]	Synthetic data	Not conducted	Behavioral verification through baseline (no-intervention) steady-state analysis	Not conducted
13	Luo et al [19]	COVID-19 and mobile phone mobility data from four Chinese cities (Guangzhou, Wuxi, Chongqing, and Ezhou)	Online estimation of time-varying infection rates with time-lag correction	Goodness-of-fit against historical curves, comparison of D-SIHR model with standard compartmental models	Impact of hospital capacity and mobility penalties on policy performance, impact of initial infection rate on generalizability

### Individual study findings

Table 4 summarizes the results of the studies included in the review. Each study is analyzed regarding its simulation setup, epidemic control outcomes, and policy evaluation.

**Table 4:** Results of the studies.

#	Study	Epidemic Outcomes	Policy evaluation
1	Shuvo et al [7]	Numerical results in epidemic control are not explicitly stated	Outperformed other DQRL algorithms in terms of average reward
2	Zhang et al [8]	Replicated real-world epidemic trends. Mask-wearing, staying home, and gathering restrictions were the most effective measures	Prioritizing economic or health factors led to stricter policies, while prioritizing a psychological factor or combining all led to more lenient ones
3	Libin et al [9]	Reduced infection prevalence by ~10% compared to the no-closures baseline	Learned near-optimal policies with stable convergence. Multi-district joint policy outperformed independent district policies
4	Zong and Luo [10]	Reduced infections and deaths. Strategies adapted to state-specific conditions	Outperformed two other algorithms with higher cumulative reward and faster convergence. Demonstrated scalability and adaptability
5	Guo et al [11].	Reduced epidemic duration, minimized lockdowns, and lowered infection peaks compared to the heuristic policies. Controlled two variants of the virus	Outperformed heuristic policies. Transformer-based mechanism with a 20-day input improved performance. Demonstrated scalability and adaptability to different variants, vaccines, and conditions
6	Hu et al [12]	Prevented a severe epidemic in 19 of 20 regions of Italy for two consecutive weeks	Improved global accumulated rewards by 119% over the baseline. *k* nearest neighbors = 1 improved the global discounted reward by 218% compared to *k* = 0. *k* > 2 showed unstable performance
7	Du et al [13]	Reduced the cumulative infections in both cities with long-term control	Outperformed baselines in reducing the cumulative number of infections. Multi-mode interventions were generally preferred over single-mode interventions. Demonstrated scalability and adaptability
8	Kompella et al [14]	Mitigated the epidemic, keeping hospital capacity under control while using more lenient restrictions	Outperformed heuristic policies by reducing infection peaks, preventing hospital overcapacity, lowering deaths, and achieving better cumulative rewards
9	Wan et al [15]	Reduced both economic losses and cumulative infections more than heuristic policies	Outperformed heuristic policies by better balancing epidemiological and economic objectives. Demonstrated robustness and adaptability
10	Ohi et al [16]	Reduced infections and deaths, while avoiding prolonged lockdowns and maintaining economic activity	The agent with a 30-day memory length achieved optimal epidemic and economic outcomes and outperformed both agents with other memory lengths and traditional cyclic lockdown strategies
11	Luo et al [17]	Contained spread while minimizing socio-economic impact. Maintained infection ratio <0.324% even for high-transmission scenarios (*R*_*0*_ = 6.5)	Hi-RICE outperformed state-of-the-art RL methods and heuristics. Decentralized R-MARL algorithm outperformed centralized RL by exploring effective policies more efficiently
12	Samadi et al [18]	Reduced average infection probability across networks under fixed budget constraints	DDPG outperformed random policies. Increasing trained agents (1–5) enhanced control
13	Luo et al [19]	Simultaneously minimized hospital strain and mobility loss in four cities. Achieved “zero new cases” while maintaining 56%−63% mobility	Outperformed RL and heuristic baselines. Entropy Weight Method was used to dynamically adjust weights in multi-objective reward function. Expert experience replay buffer allowed to prevent suboptimal strategies and improved learning efficiency

The study by Shuvo et al [7] simulates COVID-19 dynamics over 50-week episodes, with the RL agent selecting among four intervention options: full, partial, minimal lockdowns, and no action. While the model simulates different lockdown intensities and their societal impacts, the study does not provide direct epidemic outcome metrics such as infection rates or mortality reduction. Instead, outcomes are evaluated using a normalized reward function balancing health burden and intervention severity. Among the algorithms tested, N-step Deep Q-Learning (*N* = 3) consistently outperforms Deep Q-Learning, Double DQ-Learning, Deep SARSA, and Dueling DQ-Learning in terms of both normalized total reward and stability. Although epidemic-specific outcomes remain implicit, the improved reward suggests greater intervention efficiency. Additionally, the model was compared‌‌ against deterministic and random agents to demonstrate its effectiveness.

Zhang et al [8] conduct the simulation in a NetLogo environment with a 64 × 64 grid world, where 500 agents navigate daily routines, including homes, workplaces, and hospitals. The simulation spans 50 days, with the RL agent making decisions every 5 days starting from the tenth day. The sensitivity analysis indicated that mask-wearing (0.94 sensitivity), staying home (0.93), and limiting gatherings (0.92) were the most effective measures. Area lockdown (0.60) and isolation of infected individuals (0.78) were less impactful but still contributed to epidemic control. The analysis of the prioritization of various factors in the reward function shows that when prioritizing economic or health factors, the model favors strict lockdowns until infections decline to a safe level, whereas emphasizing psychological factors leads to a preference for minimal restrictions. Balancing all factors results in a more lenient approach, maintaining some restrictions while avoiding excessive lockdown measures.

Libin et al [9] present a simulation of pandemic influenza spread across Great Britain using a meta-population model of 379 interconnected administrative districts, each represented by an age-structured stochastic SEIR model. The RL agent controls weekly school closures with a limited budget. Simulations were run over 43 weeks. Two kinds of experiments were conducted. In the first one, a single-district setting was used to establish a ground truth via an exhaustive search in a deterministic version of the model. For ten demographically diverse districts and two reproductive numbers (*R*_0_ = 1.8 and 2.4), the learned policies closely matched the ground truth in reducing the proportion of the population infected under various school closure budgets (2, 4, 6 weeks), confirming that the model converges to near-optimal solutions. In the second experiment, multi-agent coordination was evaluated by learning a joint policy across 11 highly interconnected districts in the Cornwall-Devon region and comparing it to independent policies learned separately for each district and applied simultaneously. While both approaches reduced infections, the joint policy consistently achieved lower infection levels, particularly under moderate transmission (*R*_0_ = 1.8), demonstrating a clear collaborative advantage.

Zong and Luo [10] propose the Multi-Agent Recurrent Attention Actor-Critic (MARAAC) algorithm, an RL approach designed to handle time-varying epidemic environments, and apply it to simulate the spread of COVID-19 across four US states, California, Arizona, Nevada, and Utah, each with distinct demographic and economic characteristics. The simulation spans 20 weeks, with weekly updates to lockdown policies. The environment simulates daily population movement and interactions across various locations. Two experimental settings were used: in the first, each state was assigned an RL agent; in the second, RL agents managed specific location types within each state. Results show that the RL-based policy adapts effectively to state-specific conditions: California implemented prolonged strict lockdowns due to its large population, Nevada applied strict initial measures reflecting its tourism-driven exposure, while Utah maintained minimal interventions and still achieved the lowest death toll, benefiting from a small, young population. The learned policies effectively controlled infection levels and minimized deaths in all cases. The proposed algorithm outperformed two other multi-agent algorithms (MADDPG and MAAC) in terms of training stability, convergence speed, and final reward values. It also showed robust transferability when scaled to an environment with 100,000 agents.

Guo et al [11] introduce PaCAR, a pandemic control decision-making framework integrating large-scale ABM. The simulation replicates epidemic dynamics in King County, USA, and Beijing, China, utilizing over 10 million agents and runs for a maximum of 200 days per trial, with the RL agent taking action every three days. The model incorporates vaccines and non-pharmaceutical interventions. To evaluate the framework, the authors designed benchmark policies, three representing different levels of restrictions and two others simulating real-world governments’ approaches: China’s relatively strict policy and Sweden’s open policy. PaCAR framework demonstrates superior epidemic control compared to them. In King County (population of 2.23 million), without vaccines, the epidemic duration was reduced to 41 days, requiring only 6 days of lockdown, whereas other policies lasted 118–141 days with longer lockdowns. Infection peaks remained under 30 cases, far lower than the 1447–1857 in non-adaptive approaches. These numbers are slightly higher than those under strict policies, but PaCAR achieves lower economic losses. With vaccines, PaCAR controlled the Alpha variant in 41 days without lockdowns and mitigated the Delta variant in 47 days, requiring only 18 days of lockdown compared to 37–45 under other policies. In Beijing (population of 21 million), it outperformed all benchmarks, containing the Delta outbreak in 87 days with just 6 days of lockdown, whereas the strictest alternative required 59 days. PaCAR leverages DQN augmented with a Transformer-based self-attention mechanism, enabling the agent to make decisions based on historical data trends rather than single-day statistics. The analysis demonstrated that the best performance was achieved with 1–2 transformer layers and 20-day input. Shorter sequences (5 days) lacked sufficient temporal context, while longer ones (40 days) diluted relevant information.

Hu et al [12] introduce the decentralized graph-based RL using reward machines (DGRM) algorithm to simulate COVID-19 spread across Italy’s 20 regions, each modeled as a separate agent within a graph-based MDP framework. The regions interact based on fluxes of movement between them. The simulation lasts 28 days with daily discretization, and each region selects from four actions: no intervention, social distancing, inter-regional flux control, or full lockdown. Initial conditions are set such that all regions start in a severe epidemic state. The control objective is that each region would not be in a severe situation for two consecutive weeks, and avoid a long lockdown of two consecutive weeks. Epidemic outcomes show that DGRM with *k* = 1, meaning each agent’s policy and Q-function are based on local information within its 1-hop neighborhood, enables 19 of 20 regions to avoid sustained lockdowns and hospital saturation. The method outperforms the bang-bang baseline by improving the global accumulated reward by 119%, and only one region failed to meet control goals, compared to 13 under the baseline. DGRM with *k* = 1 improves global discounted reward by 218% compared to *k* = 0. Increasing *k* beyond 2 showed no significant gain due to higher learning complexity.

Du et al [13] present a simulation based on COVID-19 data from two Chinese cities, Changchun and Shanghai, which were selected due to their contrasting population densities and sizes. The simulation spans one month in each city, corresponding to real outbreaks in March-April 2022, with the RL agent making intervention decisions daily. The effectiveness of the proposed algorithm (HRL4EC) was compared to several baselines: ground truth (official intervention policies), no intervention, random, and DRL-EC (an algorithm that implements only a city lockdown). The study reports that HRL4EC consistently outperforms all baselines, achieving the lowest cumulative infection rates in both cities. Key insights include the effectiveness of moderate mobility constraints, greater temporary medical resource demand in more densely populated areas, and the benefit of low-level, sustained necessities supply to maintain compliance. Multi-mode interventions are generally preferred, but the agent sometimes switches to single-mode interventions in early or low-density outbreak conditions. In general, HRL4EC demonstrates generalizability by adapting policies appropriately across cities with different structural and demographic attributes. A sensitivity analysis reveals the model’s robustness across various reproductive numbers (*R*_0_ = 7.5 to 8.5), budget levels (80–100%), and economic-health trade-off weights.

Kompella et al [14] introduce an agent-based simulation platform modeling fine-grained human interactions across various locations in a 1,000-agent community. Each episode simulates 120 days, with the RL agent selecting one of five restriction levels daily and observing only the imperfect testing information and number of hospitalizations. The study evaluates multiple benchmark policies, including three handcrafted rule-based interventions and models of Swedish and Italian governmental strategies. Without intervention, the entire population becomes infected, and hospital capacity is exceeded early. Static policies show expected trends: higher restriction levels reduce infection peaks and deaths but increase the economic burden. The RL-optimized policy adapts to infection dynamics, keeping critical cases below capacity while minimizing restrictions. It consistently outperforms all baselines across epidemic and economic metrics, including infection peak, death rate, and cumulative reward. The policy generalizes well when tested in a larger 10,000-agent population and under reduced action frequency (every 3 or 7 days).

Wan et al [15] developed a multi-objective, model‐based framework for real-time infectious disease control, demonstrated through simulations of COVID-19 dynamics in six Chinese cities over 120 days. The RL agent selects among three intervention levels, ranging from minimal action to stringent lockdown, every seven days. The Pareto‐optimal policies derived via grid search and a DQN-based method consistently outperform baseline policies in lowering both infection counts and economic losses. The framework’s robustness is further validated through sensitivity analyses involving varying hyperparameters, employing a generalized SEIR model, and testing the model using less infectious H1N1 virus parameters. Additionally, the model produces prediction bands that reliably capture the observed epidemic curves and cost trajectories, with cross-validation yielding an average error ratio of 18%.

Ohi et al [16] present a virtual environment simulating a COVID-19 pandemic. The simulation is executed within a 2D grid world of 10,000 agents and lasts until the disease is fully mitigated. Economic contribution depends on individuals’ mobility and health, meaning that infectious and deceased individuals do not contribute to the economy. The RL agent selects movement restrictions daily across three levels: 0%, 25%, and 75% movement restrictions. Comparing agents with different memory lengths, the best-performing one with a 30-day memory achieves minimal deaths and infections while securing better daily and total economic gains. It applies initial strict lockdowns to curb the first wave and follows with cyclic and short-term lockdowns to suppress resurgence. The learned policy outperforms traditional cyclic lockdown strategies like the 7-work-7-lockdown rule in flattening infection curves.

Luo et al [17] simulate the spread of respiratory infections using an agent-based model of Shenzhen, China. The simulation involves 1.6 million agents distributed across 673 administrative communities over 150-day episodes. The study introduces the Hi-RICE framework, where RL agents make daily regional decisions to set the intensity levels for isolation, home quarantine, and community confinement. To manage the massive scale, the authors use a multi-agent reinforcement learning algorithm (MAPPO) with centralized training and decentralized execution, using LSTM layers to process temporal data. Individual-level interventions are then assigned based on a risk assessment model (I-RAM) that considers both movement history and social contact density. The framework was tested against ten baselines, including expert heuristics and state-of-the-art machine learning methods, across low, medium, and high *R*_*0*_ scenarios. Results show that Hi-RICE significantly outperforms all benchmarks in balancing infection control and economic costs. The agents demonstrate proactive behavior, applying strict measures in the early stages of an outbreak and shifting to targeted, less restrictive control as cases decline. Sensitivity analyses further confirm the model’s robustness to *R*_*0*_ uncertainty and varying levels of public compliance.

Samadi et al [18] apply a networked SIS model to simulate disease spread on weighted Erdős–Rényi graphs with 50, 75, and 100 nodes. They use DDPG algorithm to manage a continuous action space. Decentralized agents control specific subgraphs by adjusting connection weights, which represents the intensity of social distancing. Each agent operates under partial observability and a strict budget constraint. The study compares trained DDPG agents against untrained baselines across varying agent counts. Results indicate that in a 50-node network with five agents, the average infection probability was reduced from 0.728 to 0.488, while in a 100-node scenario, it decreased from 0.821 to 0.780. Sensitivity analysis shows that increasing the number of agents significantly enhances epidemic control, although the framework’s effectiveness diminishes as the network size scales up without a proportional increase in the number of agents.

Luo et al [19] use the H2-MARL framework, applied to COVID-19 control across four Chinese cities (Guangzhou, Wuxi, Chongqing, and Ezhou) using township-level meta-population models. In this environment, agents manage daily mobility quotas to balance hospital capacity against movement restrictions. A key technical innovation is the use of the Entropy Weight Method to dynamically weight these competing objectives, which helps the policy to adapt to environmental uncertainty. Furthermore, the model incorporates accumulated mobility losses with a decay factor into the state representation; this serves a role similar to sequence modeling techniques in other studies by providing the agent with long-term context regarding intervention fatigue and past policy impacts. Results showed that H2-MARL significantly outperformed heuristic and standard RL baselines, such as PPO and DQN. In Guangzhou, the framework maintained approximately 63% mobility while reducing hospital strain by 25.9% compared to existing graph-based RL approaches. The learned policies also exhibited spatial adaptability, imposing stricter restrictions on high-density urban centers while allowing greater flexibility in sparsely populated districts.

### Synthesis of results

This section synthesizes the key methodological patterns and design choices identified across the reviewed studies regarding the unique challenges of applying RL to epidemic control, such as time delays, population diversity, and data uncertainty.

#### Temporal delays.

To address the inherent non-Markovian nature of epidemic spread characterized by significant time lags, such as incubation periods and testing delays, several studies implement modifications to embed temporal dependencies into the decision-making process. One example is to incorporate sequence modeling methods, which allow agents to recognize progression trends rather than rely on single-step observations of health-state variables. For instance, study [10] integrates Gated Recurrent Units to maintain hidden states that track a 7-day history of epidemic data. Study [11] leverages a Transformer-based model with a 20-day sliding window to capture long-term dependencies. Study [16] adopts a memory-based architecture using bidirectional LSTM layers to process 30-day sequences of epidemic data, demonstrating that this length is optimal for capturing the propagation cycle of the disease. Similarly, study [17] utilizes LSTM layers and temporal “lookback windows” to link individual mobility history to delayed infection reports during latent periods.

Beyond state representation, some works employ algorithmic adjustments to reconcile actions with delayed outcomes. Study [7] addresses this by using N-step Deep Q-Learning to propagate reward information backward more effectively, ensuring the agent accounts for the cumulative future impact of a current intervention. In a different approach to reward structure, study [12] utilizes Reward Machines to define non-Markovian reward functions, enabling the agent to track high-level progress over extended periods, such as maintaining low infection levels for consecutive weeks. Other studies focus on correcting the observation gap itself. Study [14] explicitly models environmental lags within their simulator to train agents to operate effectively under partial observability. Study [15] employs model-based simulation to generate “proxy states” that estimate true infectious counts. Study [19] addresses the time-lag issue by using probabilistic adjustments for unobserved cases and includes historical impact by encoding an accumulated restriction loss with a decay factor into the state representation. Collectively, these findings indicate that incorporating temporal structure is a crucial design choice, as approaches that account for temporal dependencies and information latency report improved policy stability and performance compared to traditional Markovian approaches.

#### Spatial and demographic heterogeneity.

Effective epidemic control requires interventions tailored to the specific spatial and demographic characteristics of an outbreak, as uniform policies often impose unnecessary socio-economic burdens on low-risk areas while failing to contain localized hotspots. Capturing this heterogeneity enables context-aware decision-making.

The reviewed studies show different approaches regarding spatial heterogeneity, ranging from abstract representations to interconnected networks. Specifically, study [16] utilizes a generic 2D grid that captures micro-level movement. Meta-population [9,19] and graph-based approaches [12,18] explicitly model spatial heterogeneity by treating administrative districts or individuals as coupled nodes in a network. This allows RL agents to account for spatial interactions and develop coordinated strategies. Study [10] assigns agents for specific states, demonstrating that RL can adapt strategies to unique local profiles such as Nevada’s tourism risks or California’s large population. Studies [11,17] assign individuals to specific buildings and communities, enabling for localized risk tracking at the neighborhood level.

Regarding demographic heterogeneity, agent-based simulations [11,14,17] leverage census-grounded attributes like age, occupation, and health risk to determine agent behavior. RL agents then can evaluate how group-specific actions, such as school closures, influence the overall epidemic trajectory. In compartmental settings, study [9] stratifies the state space into age-specific compartments, allowing the agent to react to infection trends within vulnerable groups. In contrast, models relying on aggregate health variables [8,15,18,19] may overlook these sub-population nuances, potentially leading to less targeted interventions.

The capacity of an RL agent to exploit modeled heterogeneity is dictated by its action space design. Most studies utilize restriction levels that bundle multiple interventions, which simplifies the control problem but limits precision. However, more sophisticated approaches enable targeted control; for example, study [10] allows agents to allocate restrictions to specific locations like schools or retail stores, directly influencing high-contact demographic groups. Study [13] employs a hierarchical RL structure that separates high-level strategy from low-level intensity adjustments. This hierarchical approach is also seen in study [17], where regional agents determine intervention intensities for communities, which are then translated into specific individual-level restrictions. Study [19] enables spatially targeted control by managing mobility quotas between specific administrative pairs, regulating population flow to prevent the spread between townships. Finally, study [18] adjusts the weights of each network edge, which allows the agent to fine-tune the intensity of social distancing within its local neighborhood. Transitioning to high-fidelity representations and decentralized architectures is necessary to produce policies that are both epidemiologically effective and socio-economically nuanced.

#### Multi-level state representation.

State space formulation for epidemic control centers on reconciling the multi-scale mechanics of disease transmission with broader epidemiological monitoring. This challenge exists because public health policy is almost always based on population-level statistics, while an outbreak is driven by the behavior of individuals.

Most studies construct state spaces that rely heavily on macroscopic epidemiological metrics. Works that utilize compartmental models or simplified simulations define the state space exclusively through infection rates and counts [8,15,16]. Even when studies employ more complex agent-based modeling, they intentionally restrict the RL state space to aggregated data [7,10,11,14]. This abstraction is often a deliberate design choice to mimic the limited data available to real-world officials or to avoid the curse of dimensionality during training.

Sophisticated strategies for multi-level fusion attempt to capture structural determinants through spatial or demographic stratification. This is achieved through the discussed before age-specific stratification used in the study [9] and the graph-based representations employed in the study [12]. Study [13] incorporates a human contact degree matrix directly into the state representation. Finally, the recent study [17] advances this fusion by decoupling the state space into two layers: aggregated regional statistics and granular individual-level data, such as mobility patterns and contact networks.

#### Uncertainty and attribution.

In population-level epidemic control, the relationship between an intervention and its observed outcome is inherently probabilistic and confounded by external factors, such as spontaneous behavioral changes or the emergence of new viral variants. This creates a significant reward attribution challenge, as agents must distinguish whether a reduction in transmission is a direct result of their policy or a consequence of something else.

Study [10] employs counterfactual reasoning to isolate the marginal impact of specific interventions within multi-agent environments. Their MARAAC algorithm utilizes a counterfactual baseline to distinguish local policy effects from the influence of neighboring regions. Study [15] addresses parameter uncertainty by implementing a model-based framework that leverages Bayesian estimation. By accounting for uncertainty in transition model parameters rather than assuming deterministic outcomes, they generate prediction bands that provide policymakers with a visual representation of risk instead of single-point estimates. Study [17] uses temporal lookback windows to attribute risk to specific locations visited during latent periods. To reduce uncertainty from data reporting delays, study [19] introduces a time-lag correction method that uses the distribution of the serial interval to estimate under-reported infections in real-time. Previously discussed sequence modeling methods [10–12,16,17] and N-step DQN [7], which are essential for bridging temporal lags, also assist in reward attribution by linking delayed epidemiological effects to the specific interventions.

Despite these developments, current research lacks explicit risk-sensitive RL architectures designed to optimize for worst-case scenarios rather than average performance. Furthermore, most models treat population compliance as a static parameter or a psychological penalty in the reward function rather than a dynamic feedback loop. Exceptions include study [13], which models how the supply of necessities influences public cooperation, and study [19], which incorporates an “accumulated restriction loss” with a decay factor to represent behavioral fatigue and the historical impact of past interventions. Moving forward, the adoption of risk-sensitive RL architectures and the integration of dynamic behavioral models will be essential to ensure that policies remain robust under high uncertainty and shifting social compliance.

#### Interpretability.

The adoption of reinforcement learning in public health depends on providing transparent reasoning for interventions that significantly impact society. Several works address this by extracting interpretable patterns from learned behaviors to provide actionable insights. Studies [13,14,16,17] identify specific strategies like applying long initial lockdowns followed by cyclic restrictions or maintaining moderate mobility levels to balance infection control with socio-economic stability.

Several studies improve transparency through architectural choices. Reward Machines in study [12] break down complex goals into smaller, understandable stages, making the agent’s internal decision-making process more transparent. Study [15] employs threshold-based parametric policies where actions trigger at specific infection ranges, and improves trustworthiness by providing decision-support tools like Pareto-optimal trade-offs and prediction bands that allow officials to visualize epidemiological versus economic costs. In study [19], an agent can learn from established public health heuristics by using an “expert experience replay buffer.” The dynamic weighting method also enhances explainability by revealing why the agent shifts focus between hospital capacity and mobility; the weight allocation directly tracks whichever objective is currently more critical.

Another way to enhance trust is the development of realistic simulations that accurately reflect real-world dynamics [7–11,13,14,17,19]. By integrating diverse factors like vaccination rates or demographics, studies [10,11,17,19] demonstrate that RL provides more nuanced reasoning than narrow heuristic rules, such as recommending intermittent relaxations to mitigate economic damage. Despite these efforts, the issue of interpretability is not sufficiently addressed across all reviewed studies and remains a critical methodological gap in the field.

## Discussion

### Summary

The reviewed studies exhibit recurring methodological patterns and design choices that reflect how RL is currently applied in epidemic policymaking. Key trends relate to algorithm selection, epidemic modeling strategies, temporal data processing, and reward design.

All studies showed that RL-based policies outperform heuristic policies and real-world governmental strategies across key epidemiological and economic metrics. In those studies where adaptability was explicitly evaluated, the proposed models demonstrated the ability to (1) maintain performance under varying epidemic parameters and environmental conditions [8–13,15,17,19], and (2) scale effectively to larger populations and more complex environments in agent-based simulations [10,11,14]. These findings indicate that RL methods offer superior performance in epidemic mitigation and show strong generalization and robustness. This can be explained by the key strength of RL in this domain, which is its ability to navigate high-dimensional and stochastic state spaces to discover non-obvious, adaptive NPI strategies that human experts might overlook. This enables context-sensitive approaches where interventions are dynamically tuned to the specific velocity and scale of an outbreak. Conversely, a critical limitation is the sensitivity to the “sim-to-real gap,” where policies trained on idealized models may lack robustness when faced with the unpredictable behavioral shifts and structural complexities of actual populations.

This gap is directly linked to the data reliability, which determines the accuracy of the simulated environment and the resulting effectiveness of RL-trained policies. It is important since the reviewed studies utilize public health surveillance records from the COVID-19, 2009 H1N1 influenza, and SARS pandemics. Poor data quality, specifically low reporting accuracy and temporal lags, limits the agent’s ability to identify the true epidemic state. When data is sparse or delayed, the agent develops policies based on an inaccurate perception of the epidemic dynamics. This leads to miscalculated intervention timing or intensity, which can cause hospital overcapacity or prolonged socio-economic disruption in real-world applications. High data availability allows for more granular calibration of age-, demographic-, or region-specific parameters.

The introduction of sequence modeling methods is an important step towards overcoming these data-related challenges and the non-Markovian nature of epidemic control. These methods specifically address the time lags between an intervention and its epidemiological outcome, which otherwise complicate the reward attribution process. Studies using sequence-aware models report improved policy performance, suggesting that incorporating temporal structure into the state representation is a beneficial design choice. On the other hand, approaches that still rely on short-term reward signals can lead to sub-optimal decisions due to the agent’s inability to correctly relate current interventions to the delayed epidemiological effect. Future research should focus on methods related to the Credit Assignment Problem to avoid the “myopia” of policies in real conditions.

One of the most evident methodological patterns is the frequent use of Deep Q-Networks. Five out of thirteen studies employed DQN or its variants as the primary algorithm for epidemic control. Several factors may explain this preference. DQN was originally designed for environments with a limited set of discrete actions, such as the Atari games [24], making it well-suited for epidemic policymaking tasks that involve selecting from a limited set of intervention strategies. DQN offers reasonable sample efficiency, which is beneficial in computationally intensive simulations. Its relative simplicity and ease of implementation make it particularly accessible for researchers from healthcare and epidemiology. The explicit assignment of Q-values to actions enhances interpretability, which is advantageous when communicating decisions to policymakers. Finally, DQN’s prominence as one of the earliest and most widely adopted deep RL algorithms may contribute to its continued popularity. In contrast, policy-based and hybrid algorithms, like PPO and SAC, offer advantages in stability or robustness under partial observability, but often at the cost of increased complexity and training instability in small discrete settings.

Another critical design choice concerns how epidemic dynamics are modeled. The reviewed studies employ three epidemic modeling approaches: compartmental models, agent-based models, and hybrid combinations. Compartmental models offer mathematical tractability for population-level forecasting, but rely on the assumption that all individuals within a compartment have an equal probability of being infected. This assumption represents a significant limitation for fine-grained policy design, as it fails to capture localized transmission hotspots or the influence of individual mobility patterns on transmission. ABM is generally preferable for epidemic control, as it enables the representation of individual-level heterogeneity, behavioral patterns, and spatially structured interactions. By simulating these micro-level details, ABM allows RL agents to evaluate the impact of granular measures, such as localized lockdowns or specific gathering restrictions, which population-level models cannot adequately resolve. However, ABM is computationally intensive and often challenging to scale. Nevertheless, Guo et al [11] developed a simulation framework involving over 10 million agents, demonstrating that large-scale, detailed ABM is feasible for RL-based epidemic control.

The reward function is a central component in RL. It directly influences the trade-offs the agent learns to make. In eleven out of thirteen studies, the reward function is constructed to balance epidemiological outcomes with social or economic costs, reflecting the trade-offs inherent in real-world policymaking. Despite the prevalence of this design, the representation of economic loss is generally simplified. In most cases, it is incorporated indirectly by penalizing the duration or intensity of lockdowns, serving as a proxy for economic damage. Less commonly, economic loss is linked to epidemic severity, where infections or deaths reduce the agent’s reward to reflect assumed productivity loss. While economic trade-offs are central to the policy objective, their modeling remains limited in fidelity and often lacks grounding in real economic indicators.

The transition from theoretical modeling to practical deployment demands a fundamental shift beyond purely epidemiological metrics. Although RL agents demonstrate high technical performance, their long-term viability depends on how effectively they navigate social dynamics and ethical constraints. A critical gap remains in the modeling of recursive feedback loops between policy execution and public sentiment. Neglecting these behavioral dynamics, particularly the phenomenon of behavioral fatigue, risks producing mathematically optimal policies that are unsustainable in real-world contexts.

Similarly, ethical challenges are typically reduced to balancing weights in a multi-objective reward function. Beyond acknowledging mental health consequences [8] or privacy risks [11], the reviewed studies provide few mechanisms for ensuring the accountability of autonomous decision-making. Transitioning to practical deployment requires risk-sensitive architectures that treat social compliance as a dynamic state and incorporate explicit ethical constraints. RL agents should function as transparent decision-support tools rather than autonomous enforcers to maintain public trust.

### Limitations

This scoping review has several limitations that may influence the interpretation and generalizability of its findings. First, the search strategy was limited to four major academic databases and used a predefined set of keywords. Although these databases are comprehensive, relevant studies indexed elsewhere or using alternative terminology might have been omitted.

Second, the eligibility criteria focused exclusively on studies where a RL agent explicitly acts as a policymaker for non-pharmaceutical interventions. While this strict focus ensures conceptual coherence, it also excludes related applications of RL in epidemic control, such as resource allocation or crowd avoidance, that might offer transferable insights. For instance, studies focused strictly on testing might offer more sophisticated methodologies for state estimation, and pure resource allocation research could offer insights into logistical constraints and multi-objective trade-offs that are often simplified in NPI-centric works.

Third, the search strategy primarily focused on the intersection of RL and agent-based modeling. Therefore, this study is subject to a selection bias: research applying RL with compartmental models was likely omitted if the text did not include ABM-related terms. While some studies relying only on compartmental dynamics were captured, the results mainly represent literature that aligns with both reinforcement learning and agent-based modeling.

Fourth, this review did not conduct a formal critical appraisal of the included studies. Given that scoping reviews aim to map the extent and nature of evidence rather than evaluate methodological quality, this omission aligns with standard practice. However, it prevents any conclusions regarding the internal validity or empirical robustness of the included models.

Finally, heterogeneity in simulation environments, epidemic models, evaluation metrics, and inconsistencies in reporting key experimental details complicate direct comparisons. Although common trends were identified, differences in modeling assumptions and experimental setups limit the ability to generalize performance claims or compare algorithmic efficiency under unified conditions.

### Conclusions

This scoping review mapped how RL is currently employed to design non‑pharmaceutical intervention policies for epidemic control. Across thirteen studies, RL algorithms consistently surpassed heuristic strategies in reducing infections, deaths, or lockdown duration while mitigating socio‑economic disruption. Agent‑based simulations enabled granular modeling of population heterogeneity; compartmental or hybrid models offered tractability. Sequence‑aware state representations improved adaptability to dynamic epidemic conditions. Findings support the feasibility of RL‑driven policymaking frameworks when real‑world experimentation is impractical or unethical.

Nevertheless, methodological diversity limits cross‑study comparability. Economic costs are often simplified, epidemic models differ in calibration rigor, and evaluation metrics lack standardization. Few studies quantify uncertainty, assess policy robustness to data shifts, or verify transferability beyond the simulated setting.

Several research gaps should be addressed to move RL from simulation studies to practical epidemic-response applications. First, common benchmark environments and reporting standards are required to make comparative evaluations rigorous and reproducible. Second, empirically grounded economic and behavioral models should be integrated to capture broader societal trade‑offs. Third, uncertainty‑aware and probabilistic RL frameworks need to be adopted to enhance robustness to data noise and partial observability. Fourth, the more sophisticated action spaces should be developed by applying hierarchical and hybrid (discrete‑plus‑continuous) methods. These methods should decompose composite restriction levels into individually adjustable interventions and support spatially targeted interventions. Fifth, implementing counterfactual methods allows for what-if assessments of historical outbreaks to evaluate the effectiveness of specific interventions. Sixth, RL models should incorporate traditional ensemble modeling techniques to ensure that interventions remain robust across various scenarios. Finally, learned policies must be validated prospectively by deploying RL‑based decision‑support tools during live outbreaks, in collaboration with epidemiologists and public‑health agencies, to assess performance under data lags, behavioral shifts, and operational constraints.

## Supporting information

S1 Checklist PRISMA checklist. (DOCX)
