# Supplementary material for: Reinforcement learning for policymaking in epidemic control: A scoping review
Source: PLoS One. 2026 Jun 8;21(6):e0351176. doi: 10.1371/journal.pone.0351176 (PMC13245796; doi:10.1371/journal.pone.0351176)
Supplement: Attachment — Submitted filename: Comments for RL.pdf [file pone.0351176.s002.pdf]

This manuscript presents a timely and valuable scoping review on the application of Reinforcement Learning (RL) in epidemic control policymaking. The topic is of high relevance, especially in the post-COVID era, as the public health community seeks more adaptive and data-driven intervention strategies. The review is generally well-structured, follows PRISMA-ScR guidelines, and identifies a clear gap in the literature.

However, the manuscript requires major revisions before it can be considered for publication. The primary concerns revolve around the justification of the scope, the depth of methodological critique from an epidemiological perspective, and the clarity of public health implications. Addressing these points will significantly strengthen the manuscript's contribution to both the computer science and public health fields.

Major Comments

1. The decision to limit the review to studies where an RL agent explicitly acts as a policymaker for NPIs is understandable but needs stronger justification (P.4, L.81-82). The authors should elaborate on why related applications like resource allocation (e.g., ICU beds, vaccines) and testing strategies were excluded, as these are critical components of epidemic control policy and often involve similar RL techniques. A brief discussion on the potential insights lost by this narrow focus would strengthen the manuscript.

2. The application of RL to population-level epidemic control presents unique challenges that are less pronounced in other domains like clinical ICU management. The current review would be significantly strengthened by a critical analysis of how the included studies address (or fail to address) these core epidemiological complexities:

(1) Temporal Delays: The effects of NPIs (e.g., lockdowns) on case numbers and hospitalizations are subject to significant time lags (incubation period, testing delays, etc.). RL agents operating on a short-term reward signal may struggle with this inherent non-Markovian nature. The review should analyze which studies explicitly account for these delays and discuss this as a key challenge.

(2) Spatio-Temporal and Demographic Heterogeneity: Intervention effects are not uniform. They vary by geography, time, and sub-population (e.g., age, occupation). The review should critically assess whether the state and action spaces in the included studies are designed to capture this heterogeneity. For instance, do studies allow for spatially-targeted interventions, or do they assume homogeneous national-level policies? Is the population stratified in the state representation?

(3) Multi-Level State Representation: An effective state space for public health policy likely requires a fusion of aggregated, population-level statistics (e.g., regional case counts) and individual-level determinants (e.g., contact patterns, age structure, mobility networks). The review should evaluate the sophistication of the state spaces used and whether they reflect this multi-level reality.

(4) Uncertainty and Attribution: In population-level interventions, the link between an action and an outcome is probabilistic and confounded by numerous external factors (e.g., voluntary behavior change, new variants). An agent might implement a lockdown, but a subsequent drop in cases could be due to other reasons, and vice-versa. The review should discuss the challenge of reward attribution under uncertainty and whether any studies employ methods like counterfactual reasoning or robust/risk-sensitive RL to address it. A forward-looking discussion on how future work could tackle this is essential.

3. The search was conducted exclusively in computer science and multidisciplinary databases (IEEE, ACM, Scopus, ScienceDirect) (P.6, L.125-128). It is highly likely that relevant work has been published in public health, epidemiology, and medical informatics journals (e.g., journals indexed in PubMed/MEDLINE). This constitutes a potential selection bias. The authors must either justify this database selection robustly or, preferably, repeat the search to include PubMed/MEDLINE to ensure comprehensive coverage.

4. The search strategy explicitly included terms related to "Agent-Based Modeling" (ABM) but did not include analogous terms for "compartmental models" (e.g., "SEIR model," "SIR model," "compartmental model," "deterministic model") (P.7, L.131-144). Given that the review itself identifies compartmental and hybrid

models as common approaches (P.29-30, L.547-549), this constitutes a potential selection bias in the retrieved literature. It is plausible that studies using compartmental models with RL, which describe their work using epidemiological rather than computer science terminology, were missed. The authors must acknowledge this as a significant limitation of their search strategy and justify the original decision or, ideally, perform a supplementary search to ensure key studies using compartmental models were not inadvertently excluded.

5. The search was conducted between 2014 and 2024 (P.5, L.99), and the current date is November 2025. A significant 11-month gap has since passed in a rapidly evolving field. To ensure the review's conclusions reflect the current state-of-the-art, the authors must update their literature search to include studies published up to at least mid-2025.

6. The review descriptively lists the types of models used (compartmental, ABM, hybrid) (P.13, L.240-261) but lacks a critical appraisal of their epidemiological validity. For instance:

(1) How were key parameters (e.g., R_0, serial interval, contact rates) sourced and justified in the included studies? Were they based on empirical data or assumed?

(2) The manuscript mentions "inconsistent calibration rigor" (P.3, L.47) but does not elaborate. A more detailed analysis of the calibration and validation methods used across studies is needed (e.g., as partially mentioned in Table 3). For example, which studies calibrated to real outbreak data? Which performed out-of-sample validation or sensitivity analysis on epidemiological parameters? A table summarizing these validation aspects would be highly informative.

(3) The assumption of homogeneous mixing in compartmental models is a significant limitation for fine-grained policy design (P.13, L.245-248). This critical weakness should be discussed more prominently, explaining why ABMs are often preferred for this task and the trade-offs involved (P.30, L.549-554).

7. A major barrier to the adoption of AI/RL in public health is the "black box" problem. The review briefly mentions "interpretability" in the context of DQN (P.29, L.539-541) but does not sufficiently address this critical issue. The authors should add

a dedicated paragraph discussing the interpretability and explainability of the RL policies in the included studies. Were the learned policies analyzed to provide insights (e.g., "lockdown is triggered when hospitalizations exceed X threshold")? How can these models be made trustworthy and actionable for public health officials?

8. The conclusion calls for prospective validation (P.33, L.630-632). This should be expanded into a discussion on the practical implementation challenges, such as:

(1) Data Latency: Real-world data (cases, hospitalizations) is always delayed. How do these models handle partial and delayed observability?

(2) Behavioral Adaptation: Human behavior changes in response to both the pandemic and the policies themselves. How do these RL models account for this dynamic feedback?

(3) Ethical Considerations: The use of autonomous systems to enforce restrictive policies (like lockdowns) raises significant ethical concerns that should be acknowledged.

Minor Comments

Abstract: The sentence "Across various settings, RL policies outperform heuristic, rule-based, and historical baselines..." (P.3, L.41-43) is a strong claim. Consider qualifying it with "in simulation" or "according to the reviewed studies," to maintain scientific caution.

Table 1: The column "Epidemic Dynamics" could be more precise. For example, "SEIHRD + ABM" should be clarified as "Hybrid (SEIHRD with ABM-governed interactions)" to distinguish it from a pure ABM.

Future Directions: Consider adding counterfactual analysis (using trained RL models to run "what-if" scenarios against historical outbreaks) and integration with ensemble modeling (combining RL with traditional epidemiological ensemble forecasts to improve robustness).

Overall, this manuscript addresses a highly important and interdisciplinary topic. With the proposed revisions, particularly a more critical epidemiological perspective

on model validation and a deeper discussion of implementation challenges in public health, it has the potential to become a significant reference paper for both RL practitioners and epidemiologists interested in computational methods for policy design. I look forward to seeing a revised version.
