# Supplementary material for: Reinforcement learning for policymaking in epidemic control: A scoping review
Source: PLoS One. 2026 Jun 8;21(6):e0351176. doi: 10.1371/journal.pone.0351176 (PMC13245796; doi:10.1371/journal.pone.0351176)
Supplement: Attachment — Submitted filename: Response to Reviewers.docx [file pone.0351176.s003.docx]

# Response to Reviewers

Dear Reviewers**,**

We would like to thank you for your time and the constructive feedback provided for our manuscript. We have carefully addressed each comment, and our point-by-point responses, along with details of the corresponding changes made to the manuscript, are provided below. Please note that all line numbers in our responses refer to the “Revised Manuscript with Track Changes” file.

# Reply to comments made by Reviewer #1

## Comment 1

Line 187: The paper does not display Figure 1 (PRISMA flow diagram). Please insert the PRISMA flow diagram here. If the author has placed Figure 1 in the Appendix, please remove the caption 'Fig 1. PRISMA flow diagram' from the main text.

## Response

Following the PLOS ONE submission guidelines, figures must not be included in the main manuscript file but submitted as individual files, with captions placed immediately after the paragraph of the first citation. Figure 1 (PRISMA flow diagram) was submitted as a separate file during the initial submission.

## Comment 2

Please add more explanation about the key challenges and future research directions for implementing RL-based policymaking in real-world epidemic control scenarios.

## Response

We have added a dedicated Synthesis of results section (L. 655) that provides a thematic analysis of key challenges, including temporal delays, spatial and demographic heterogeneity, multi-level state representation, uncertainty and attribution, and interpretability. The Summary and Conclusions sections were expanded to include discussion on socio-ethical barriers (L. 924-939) and future research directions such as counterfactual analysis and ensemble modeling (L. 1002-1006).

## Comment 3

Please add more explanation about the strengths and limitations of RL algorithms in epidemic policymaking.

## Response

The Summary section has been expanded to explicitly detail the strengths of RL, and the “sim-to-real” gap and data reliability limitations (L. 837-856). Additionally, the black-box nature of RL models and its implications for public health governance were addressed in the dedicated Interpretability subsection within the Synthesis of results (L. 796).

## Comment 4

Please add an explanation of the data sources typically used to train RL models for epidemic control, and discuss how data quality and availability impact performance

## Response

We have added a new table (L. 419) regarding calibration and validation methods with a dedicated “Data source” column, which specifies the datasets and geographical origins of the data used across the reviewed studies. To address the impact of data quality and availability, a new paragraph was added to the Summary section (L. 847-856). Additionally, the paragraph concerning sequence modeling was repositioned and revised (L. 858-868) to serve as a continuation of this discussion, illustrating how such architectures mitigate data-related challenges such as temporal lags and noise.

## Comment 5

The structure of the paper should be improved to enhance clarity and better organize the content.

## Response

The paper is structured in strict accordance with the PRISMA Extension for Scoping Reviews (PRISMA-ScR) guidelines. We believe this organization provides the necessary clarity and follows the established reporting standards for this type of research.

# Reply to comments made by Reviewer #2

## Comment 1

The decision to limit the review to studies where an RL agent explicitly acts as a policymaker for NPIs is understandable but needs stronger justification (P.4, L.81-82). The authors should elaborate on why related applications like resource allocation (e.g., ICU beds, vaccines) and testing strategies were excluded, as these are critical components of epidemic control policy and often involve similar RL techniques. A brief discussion on the potential insights lost by this narrow focus would strengthen the manuscript.

## Response

We agree with the reviewer that resource allocation and testing strategies are critical components of epidemic control. However, we chose to focus exclusively on NPI policymaking because it involves fundamentally different objective functions and methodologies compared to logistical optimization or state estimation. To address the reviewer’s concern, we have added the justification in the Methods section (L. 127-135) and added a dedicated discussion in the Limitations section (L. 951-954).

## Comment 2

The application of RL to population-level epidemic control presents unique challenges that are less pronounced in other domains like clinical ICU management. The current review would be significantly strengthened by a critical analysis of how the included studies address (or fail to address) these core epidemiological complexities:

(1) Temporal Delays: The effects of NPIs (e.g., lockdowns) on case numbers and hospitalizations are subject to significant time lags (incubation period, testing delays, etc.). RL agents operating on a short-term reward signal may struggle with this inherent non-Markovian nature. The review should analyze which studies explicitly account for these delays and discuss this as a key challenge.

(2) Spatio-Temporal and Demographic Heterogeneity: Intervention effects are not uniform. They vary by geography, time, and sub-population (e.g., age, occupation). The review should critically assess whether the state and action spaces in the included studies are designed to capture this heterogeneity. For instance, do studies allow for spatially-targeted interventions, or do they assume homogeneous national-level policies? Is the population stratified in the state representation?

(3) Multi-Level State Representation: An effective state space for public health policy likely requires a fusion of aggregated, population-level statistics (e.g., regional case counts) and individual-level determinants (e.g., contact patterns, age structure, mobility networks). The review should evaluate the sophistication of the state spaces used and whether they reflect this multi-level reality.

(4) Uncertainty and Attribution: In population-level interventions, the link between an action and an outcome is probabilistic and confounded by numerous external factors (e.g., voluntary behavior change, new variants). An agent might implement a lockdown, but a subsequent drop in cases could be due to other reasons, and vice-versa. The review should discuss the challenge of reward attribution under uncertainty and whether any studies employ methods like counterfactual reasoning or robust/risk-sensitive RL to address it. A forward-looking discussion on how future work could tackle this is essential.

## Response

We have added a new Synthesis of results section (L. 655) to address this request, providing a comparative analysis of how the reviewed studies manage the four epidemiological complexities mentioned. To ensure structural clarity and avoid duplication, we have updated the Study characteristics (L. 366-374) and Summary (L. 903-910) sections.

## Comment 3

The search was conducted exclusively in computer science and multidisciplinary databases (IEEE, ACM, Scopus, ScienceDirect) (P.6, L.125-128). It is highly likely that relevant work has been published in public health, epidemiology, and medical informatics journals (e.g., journals indexed in PubMed/MEDLINE). This constitutes a potential selection bias. The authors must either justify this database selection robustly or, preferably, repeat the search to include PubMed/MEDLINE to ensure comprehensive coverage.

## Response

Scopus was chosen as the primary source specifically because it is a comprehensive multidisciplinary database that extensively covers the journals indexed in PubMed/MEDLINE. By combining it with ScienceDirect, we ensured broad coverage across both technical and medical domains. Given the high degree of overlap between Scopus and PubMed, we are confident that the current search results provide a representative map of the research landscape without significant selection bias.

## Comment 4

The search strategy explicitly included terms related to "Agent-Based Modeling" (ABM) but did not include analogous terms for "compartmental models" (e.g., "SEIR model," "SIR model," "compartmental model," "deterministic model") (P.7, L.131-144). Given that the review itself identifies compartmental and hybrid models as common approaches (P.29-30, L.547-549), this constitutes a potential selection bias in the retrieved literature. It is plausible that studies using compartmental models with RL, which describe their work using epidemiological rather than computer science terminology, were missed. The authors must acknowledge this as a significant limitation of their search strategy and justify the original decision or, ideally, perform a supplementary search to ensure key studies using compartmental models were not inadvertently excluded.

## Response

We agree that requiring agent-based terminology in the search string introduced a selection bias. While our criteria allowed for studies without ABM, the search terms likely excluded studies with pure compartmental models. To address this problem, we updated the Limitations section (L. 956-961) to acknowledge that RL studies using compartmental models without ABM terms were likely missed.

## Comment 5

The search was conducted between 2014 and 2024 (P.5, L.99), and the current date is November 2025. A significant 11-month gap has since passed in a rapidly evolving field. To ensure the review's conclusions reflect the current state-of-the-art, the authors must update their literature search to include studies published up to at least mid-2025

## Response

We have updated our literature search to include studies published up to the end of 2025. This updated search yielded 138 additional records. After removing duplicates and screening titles and abstracts, 3 new studies met the inclusion criteria and were added to the review, bringing the total number of analyzed papers to 13. We have updated the entire manuscript to incorporate these new findings.

## Comment 6

The review descriptively lists the types of models used (compartmental, ABM, hybrid) (P.13, L.240-261) but lacks a critical appraisal of their epidemiological validity. For instance:

(1) How were key parameters (e.g., R0, serial interval, contact rates) sourced and justified in the included studies? Were they based on empirical data or assumed?

(2) The manuscript mentions "inconsistent calibration rigor" (P.3, L.47) but does not elaborate. A more detailed analysis of the calibration and validation methods used across studies is needed (e.g., as partially mentioned in Table 3). For example, which studies calibrated to real outbreak data? Which performed out-of-sample validation or sensitivity analysis on epidemiological parameters? A table summarizing these validation aspects would be highly informative.

(3) The assumption of homogeneous mixing in compartmental models is a significant limitation for fine-grained policy design (P.13, L.245-248). This critical weakness should be discussed more prominently, explaining why ABMs are often preferred for this task and the trade-offs involved (P.30, L.549-554).

## Response

Regarding Comment 6.1, we have added a new Epidemiological validity subsection to the Study characteristics section (L. 376) to address how researchers justified key parameters like R0 and disease progression rates. Regarding Comment 6.2, this new section also includes a general analytical paragraph and a detailed Calibration and validation methods table summarizing data sources, calibration methods, validation methods, and sensitivity analyses for each study (L. 408-420). This table explicitly confirms the inconsistent calibration rigor across the reviewed studies. Redundant validation information was removed from the Results of the studies table and from texts in the Individual study findings section (L. 421) to avoid duplication. Regarding Comment 6.3, we expanded the modeling discussion in the Summary section (L. 886-901).

## Comment 7

A major barrier to the adoption of AI/RL in public health is the "black box" problem. The review briefly mentions "interpretability" in the context of DQN (P.29,L.539-541) but does not sufficiently address this critical issue. The authors should add a dedicated paragraph discussing the interpretability and explainability of the RL policies in the included studies. Were the learned policies analyzed to provide insights (e.g., "lockdown is triggered when hospitalizations exceed X threshold")? How can these models be made trustworthy and actionable for public health officials?

## Response

We have added a dedicated Interpretability subsection to the Synthesis of results section (L. 796) to describe how included studies address the “black box” problem and summarize the methods used to make learned policies transparent and actionable for public health officials.

## Comment 8

The conclusion calls for prospective validation (P.33, L.630-632). This should be expanded into a discussion on the practical implementation challenges, such as:

(1) Data Latency: Real-world data (cases, hospitalizations) is always delayed. How do these models handle partial and delayed observability?

(2) Behavioral Adaptation: Human behavior changes in response to both the pandemic and the policies themselves. How do these RL models account for this dynamic feedback?

(3) Ethical Considerations: The use of autonomous systems to enforce restrictive policies (like lockdowns) raises significant ethical concerns that should be acknowledged.

## Response

Regarding data latency, this challenge is already addressed in the Temporal Delays subsection (L. 660), following the response to Comment 2, and in the corresponding Summary paragraph (L. 858-868), which discusses sequence modeling techniques. Since these sections specifically detail how models handle delayed observability and non-Markovian dynamics, no additional text was added to avoid redundancy. For the points on behavioral adaptation and ethical considerations, a new paragraph has been added to the Summary section (L. 924-939).

## Minor Comments

Abstract: The sentence "Across various settings, RL policies outperform heuristic, rule-based, and historical baselines..." (P.3, L.41-43) is a strong claim. Consider qualifying it with "in simulation" or "according to the reviewed studies," to maintain scientific caution.

Table 1: The column "Epidemic Dynamics" could be more precise. For example, "SEIHRD + ABM" should be clarified as "Hybrid (SEIHRD with ABM-governed interactions)" to distinguish it from a pure ABM.

Future Directions: Consider adding counterfactual analysis (using trained RL models to run "what-if" scenarios against historical outbreaks) and integration with ensemble modeling (combining RL with traditional epidemiological ensemble forecasts to improve robustness).

## Response

We updated the Abstract with the phrase “according to the reviewed studies” (L. 43). Table 1 (L. 205) was updated to provide more precise descriptions about epidemic modeling, which included recategorizing studies Zong and Luo (2022) and Kompella et al (2020) from Hybrid to pure ABM. This correction was made after a deeper technical review confirmed that their internal SEIR-like structures describe individual agent stochastic transitions rather than population-level compartmental flows. The Epidemic dynamics modeling subsection (L. 251) was updated to reflect this correction. The Conclusions section was expanded to include two mentioned research directions (L. 1002-1006).
