# Supplementary material for: Reinforcement learning for policymaking in epidemic control: A scoping review
Source: PLoS One. 2026 Jun 8;21(6):e0351176. doi: 10.1371/journal.pone.0351176 (PMC13245796; doi:10.1371/journal.pone.0351176)
Supplement: Peer review history

# Peer review: Decision Letter 0

**DOI:** 10.1371/journal.pone.0351176.r001

**Authors:** Siew Ann Cheong

10 Dec 2025

PONE-D-25-35802

Reinforcement learning for policymaking in epidemic control: a scoping review

PLOS One

Dear Dr. Bolshov,

Thank you for submitting your manuscript to PLOS ONE. After careful consideration, we feel that it has merit but does not fully meet PLOS ONE’s publication criteria as it currently stands. Therefore, we invite you to submit a revised version of the manuscript that addresses the points raised during the review process.

Both reviewers feel that there are serious shortcomings to the manuscript. In particular, there is a lack of comparison against other machine learning approaches for epidemic management. Please address the comments raised by both reviewers to improve the manuscript.

Please submit your revised manuscript by Jan 24 2026 11:59PM. If you will need more time than this to complete your revisions, please reply to this message or contact the journal office at plosone@plos.org. When you're ready to submit your revision, log on to https://www.editorialmanager.com/pone/ and select the 'Submissions Needing Revision' folder to locate your manuscript file.

Please include the following items when submitting your revised manuscript:

- A rebuttal letter that responds to each point raised by the academic editor and reviewer(s). You should upload this letter as a separate file labeled 'Response to Reviewers'.
- A marked-up copy of your manuscript that highlights changes made to the original version. You should upload this as a separate file labeled 'Revised Manuscript with Track Changes'.
- An unmarked version of your revised paper without tracked changes. You should upload this as a separate file labeled 'Manuscript'.

If you would like to make changes to your financial disclosure, please include your updated statement in your cover letter. Guidelines for resubmitting your figure files are available below the reviewer comments at the end of this letter.

If applicable, we recommend that you deposit your laboratory protocols in protocols.io to enhance the reproducibility of your results. Protocols.io assigns your protocol its own identifier (DOI) so that it can be cited independently in the future. For instructions see: https://journals.plos.org/plosone/s/submission-guidelines#loc-laboratory-protocols. Additionally, PLOS ONE offers an option for publishing peer-reviewed Lab Protocol articles, which describe protocols hosted on protocols.io. Read more information on sharing protocols at https://plos.org/protocols?utm_medium=editorial-email&utm_source=authorletters&utm_campaign=protocols.

We look forward to receiving your revised manuscript.

Kind regards,

Siew Ann Cheong, Ph.D.

Academic Editor

PLOS One

Journal requirements:

When submitting your revision, we need you to address these additional requirements.

1. Please ensure that your manuscript meets PLOS ONE's style requirements, including those for file naming. The PLOS ONE style templates can be found at

https://journals.plos.org/plosone/s/file?id=wjVg/PLOSOne_formatting_sample_main_body.pdf and

https://journals.plos.org/plosone/s/file?id=ba62/PLOSOne_formatting_sample_title_authors_affiliations.pdf

2. Please amend either the abstract on the online submission form (via Edit Submission) or the abstract in the manuscript so that they are identical.

If the reviewer comments include a recommendation to cite specific previously published works, please review and evaluate these publications to determine whether they are relevant and should be cited. There is no requirement to cite these works unless the editor has indicated otherwise.

[Note: HTML markup is below. Please do not edit.]

Reviewers' comments:

Reviewer's Responses to Questions

**Comments to the Author**

1. Is the manuscript technically sound, and do the data support the conclusions?

The manuscript must describe a technically sound piece of scientific research with data that supports the conclusions. Experiments must have been conducted rigorously, with appropriate controls, replication, and sample sizes. The conclusions must be drawn appropriately based on the data presented.

Reviewer #1: No

Reviewer #2: Yes

**********

2. Has the statistical analysis been performed appropriately and rigorously?

Reviewer #1: No

Reviewer #2: N/A

**********

3. Have the authors made all data underlying the findings in their manuscript fully available?

The PLOS Data policy requires authors to make all data underlying the findings described in their manuscript fully available without restriction, with rare exception (please refer to the Data Availability Statement in the manuscript PDF file). The data should be provided as part of the manuscript or its supporting information, or deposited to a public repository. For example, in addition to summary statistics, the data points behind means, medians and variance measures should be available. If there are restrictions on publicly sharing data—e.g. participant privacy or use of data from a third party—those must be specified.

Reviewer #1: No

Reviewer #2: Yes

**********

4. Is the manuscript presented in an intelligible fashion and written in standard English?

PLOS ONE does not copyedit accepted manuscripts, so the language in submitted articles must be clear, correct, and unambiguous. Any typographical or grammatical errors should be corrected at revision, so please note any specific errors here.

Reviewer #1: Yes

Reviewer #2: Yes

**********

5. Review Comments to the Author

Please use the space provided to explain your answers to the questions above. You may also include additional comments for the author, including concerns about dual publication, research ethics, or publication ethics. (Please upload your review as an attachment if it exceeds 20,000 characters)

Reviewer #1: The authors reviewed peer-reviewed studies (2014–2024, English) that applied deep RL to select NPIs. A total of 27 studies were retrieved from IEEE Xplore, ACM Digital Library, ScienceDirect, and Scopus, searched on December 12, 2024. However, there are several problems that should be addressed:

1. Line 187: The paper does not display Figure 1 (PRISMA flow diagram). Please insert the PRISMA flow diagram here. If the author has placed Figure 1 in the Appendix, please remove the caption 'Fig 1. PRISMA flow diagram' from the main text.

2. Please add more explanation about the key challenges and future research directions for implementing RL-based policymaking in real-world epidemic control scenarios.

3. Please add more explanation about the strengths and limitations of RL algorithms in epidemic policymaking.

4. Please add an explanation of the data sources typically used to train RL models for epidemic control, and discuss how data quality and availability impact performance

5. The structure of the paper should be improved to enhance clarity and better organize the content.

Reviewer #2: This manuscript presents a timely and valuable scoping review on the application of Reinforcement Learning (RL) in epidemic control policymaking. The topic is of high relevance, especially in the post-COVID era, as the public health community seeks more adaptive and data-driven intervention strategies. The review is generally well-structured, follows PRISMA-ScR guidelines, and identifies a clear gap in the literature. However, the manuscript requires major revisions before it can be considered for publication. Therefore, I have uploaded my review as an attachment.

**********

6. PLOS authors have the option to publish the peer review history of their article (what does this mean?). If published, this will include your full peer review and any attached files.

If you choose “no”, your identity will remain anonymous but your review may still be made public.

**Do you want your identity to be public for this peer review?** For information about this choice, including consent withdrawal, please see our Privacy Policy.

Reviewer #1: No

Reviewer #2: No

**********

[NOTE: If reviewer comments were submitted as an attachment file, they will be attached to this email and accessible via the submission site. Please log into your account, locate the manuscript record, and check for the action link "View Attachments". If this link does not appear, there are no attachment files.]

To ensure your figures meet our technical requirements, please review our figure guidelines: https://journals.plos.org/plosone/s/figures

You may also use PLOS’s free figure tool, NAAS, to help you prepare publication quality figures: https://journals.plos.org/plosone/s/figures#loc-tools-for-figure-preparation.

NAAS will assess whether your figures meet our technical requirements by comparing each figure against our figure specifications.

Attachment Submitted filename: Comments for RL.pdf

# Author comment: Author response to Decision Letter 1

**DOI:** 10.1371/journal.pone.0351176.r002

23 Jan 2026

Dear Reviewers,

We would like to thank you for your time and the constructive feedback provided for our manuscript. We have carefully addressed each comment, and our point-by-point responses, along with details of the corresponding changes made to the manuscript, are provided below. Please note that all line numbers in our responses refer to the “Revised Manuscript with Track Changes” file.

Reply to comments made by Reviewer #1

Comment 1

Line 187: The paper does not display Figure 1 (PRISMA flow diagram). Please insert the PRISMA flow diagram here. If the author has placed Figure 1 in the Appendix, please remove the caption 'Fig 1. PRISMA flow diagram' from the main text.

Response

Following the PLOS ONE submission guidelines, figures must not be included in the main manuscript file but submitted as individual files, with captions placed immediately after the paragraph of the first citation. Figure 1 (PRISMA flow diagram) was submitted as a separate file during the initial submission.

Comment 2

Please add more explanation about the key challenges and future research directions for implementing RL-based policymaking in real-world epidemic control scenarios.

Response

We have added a dedicated Synthesis of results section (L. 655) that provides a thematic analysis of key challenges, including temporal delays, spatial and demographic heterogeneity, multi-level state representation, uncertainty and attribution, and interpretability. The Summary and Conclusions sections were expanded to include discussion on socio-ethical barriers (L. 924-939) and future research directions such as counterfactual analysis and ensemble modeling (L. 1002-1006).

Comment 3

Please add more explanation about the strengths and limitations of RL algorithms in epidemic policymaking.

Response

The Summary section has been expanded to explicitly detail the strengths of RL, and the “sim-to-real” gap and data reliability limitations (L. 837-856). Additionally, the black-box nature of RL models and its implications for public health governance were addressed in the dedicated Interpretability subsection within the Synthesis of results (L. 796).

Comment 4

Please add an explanation of the data sources typically used to train RL models for epidemic control, and discuss how data quality and availability impact performance

Response

We have added a new table (L. 419) regarding calibration and validation methods with a dedicated “Data source” column, which specifies the datasets and geographical origins of the data used across the reviewed studies. To address the impact of data quality and availability, a new paragraph was added to the Summary section (L. 847-856). Additionally, the paragraph concerning sequence modeling was repositioned and revised (L. 858-868) to serve as a continuation of this discussion, illustrating how such architectures mitigate data-related challenges such as temporal lags and noise.

Comment 5

The structure of the paper should be improved to enhance clarity and better organize the content.

Response

The paper is structured in strict accordance with the PRISMA Extension for Scoping Reviews (PRISMA-ScR) guidelines. We believe this organization provides the necessary clarity and follows the established reporting standards for this type of research.

Reply to comments made by Reviewer #2

Comment 1

The decision to limit the review to studies where an RL agent explicitly acts as a policymaker for NPIs is understandable but needs stronger justification (P.4, L.81-82). The authors should elaborate on why related applications like resource allocation (e.g., ICU beds, vaccines) and testing strategies were excluded, as these are critical components of epidemic control policy and often involve similar RL techniques. A brief discussion on the potential insights lost by this narrow focus would strengthen the manuscript.

Response

We agree with the reviewer that resource allocation and testing strategies are critical components of epidemic control. However, we chose to focus exclusively on NPI policymaking because it involves fundamentally different objective functions and methodologies compared to logistical optimization or state estimation. To address the reviewer’s concern, we have added the justification in the Methods section (L. 127-135) and added a dedicated discussion in the Limitations section (L. 951-954).

Comment 2

The application of RL to population-level epidemic control presents unique challenges that are less pronounced in other domains like clinical ICU management. The current review would be significantly strengthened by a critical analysis of how the included studies address (or fail to address) these core epidemiological complexities:

(1) Temporal Delays: The effects of NPIs (e.g., lockdowns) on case numbers and hospitalizations are subject to significant time lags (incubation period, testing delays, etc.). RL agents operating on a short-term reward signal may struggle with this inherent non-Markovian nature. The review should analyze which studies explicitly account for these delays and discuss this as a key challenge.

(2) Spatio-Temporal and Demographic Heterogeneity: Intervention effects are not uniform. They vary by geography, time, and sub-population (e.g., age, occupation). The review should critically assess whether the state and action spaces in the included studies are designed to capture this heterogeneity. For instance, do studies allow for spatially-targeted interventions, or do they assume homogeneous national-level policies? Is the population stratified in the state representation?

(3) Multi-Level State Representation: An effective state space for public health policy likely requires a fusion of aggregated, population-level statistics (e.g., regional case counts) and individual-level determinants (e.g., contact patterns, age structure, mobility networks). The review should evaluate the sophistication of the state spaces used and whether they reflect this multi-level reality.

(4) Uncertainty and Attribution: In population-level interventions, the link between an action and an outcome is probabilistic and confounded by numerous external factors (e.g., voluntary behavior change, new variants). An agent might implement a lockdown, but a subsequent drop in cases could be due to other reasons, and vice-versa. The review should discuss the challenge of reward attribution under uncertainty and whether any studies employ methods like counterfactual reasoning or robust/risk-sensitive RL to address it. A forward-looking discussion on how future work could tackle this is essential.

Response

We have added a new Synthesis of results section (L. 655) to address this request, providing a comparative analysis of how the reviewed studies manage the four epidemiological complexities mentioned. To ensure structural clarity and avoid duplication, we have updated the Study characteristics (L. 366-374) and Summary (L. 903-910) sections.

Comment 3

The search was conducted exclusively in computer science and multidisciplinary databases (IEEE, ACM, Scopus, ScienceDirect) (P.6, L.125-128). It is highly likely that relevant work has been published in public health, epidemiology, and medical informatics journals (e.g., journals indexed in PubMed/MEDLINE). This constitutes a potential selection bias. The authors must either justify this database selection robustly or, preferably, repeat the search to include PubMed/MEDLINE to ensure comprehensive coverage.

Response

Scopus was chosen as the primary source specifically because it is a comprehensive multidisciplinary database that extensively covers the journals indexed in PubMed/MEDLINE. By combining it with ScienceDirect, we ensured broad coverage across both technical and medical domains. Given the high degree of overlap between Scopus and PubMed, we are confident that the current search results provide a representative map of the research landscape without significant selection bias.

Comment 4

The search strategy explicitly included terms related to "Agent-Based Modeling" (ABM) but did not include analogous terms for "compartmental models" (e.g., "SEIR model," "SIR model," "compartmental model," "deterministic model") (P.7, L.131-144). Given that the review itself identifies compartmental and hybrid models as common approaches (P.29-30, L.547-549), this constitutes a potential selection bias in the retrieved literature. It is plausible that studies using compartmental models with RL, which describe their work using epidemiological rather than computer science terminology, were missed. The authors must acknowledge this as a significant limitation of their search strategy and justify the original decision or, ideally, perform a supplementary search to ensure key studies using compartmental models were not inadvertently excluded.

Response

We agree that requiring agent-based terminology in the search string introduced a selection bias. While our criteria allowed for studies without ABM, the search terms likely excluded studies with pure compartmental models. To address this problem, we updated the Limitations section (L. 956-961) to acknowledge that RL studies using compartmental models without ABM terms were likely missed.

Comment 5

The search was conducted between 2014 and 2024 (P.5, L.99), and the current date is November 2025. A significant 11-month gap has since passed in a rapidly evolving field. To ensure the review's conclusions reflect the current state-of-the-art, the authors must update their literature search to include studies published up to at least mid-2025

Response

We have updated our literature search to include studies published up to the end of 2025. This updated search yielded 138 additional records. After removing duplicates and screening titles and abstracts, 3 new studies met the inclusion criteria and were added to the review, bringing the total number of analyzed papers to 13. We have updated the entire manuscript to incorporate these new findings.

Comment 6

The review descriptively lists the types of models used (compartmental, ABM, hybrid) (P.13, L.240-261) but lacks a critical appraisal of their epidemiological validity. For instance:

(1) How were key parameters (e.g., R0, serial interval, contact rates) sourced and justified in the included studies? Were they based on empirical data or assumed?

(2) The manuscript mentions "inconsistent calibration rigor" (P.3, L.47) but does not elaborate. A more detailed analysis of the calibration and validation methods used across studies is needed (e.g., as partially mentioned in Table 3). For example, which studies calibrated to real outbreak data? Which performed out-of-sample validation or sensitivity analysis on epidemiological parameters? A table summarizing these validation aspects would be highly informative.

(3) The assumption of homogeneous mixing in compartmental models is a significant limitation for fine-grained policy design (P.13, L.245-248). This critical weakness should be discussed more prominently, explaining why ABMs are often preferred for this task and the trade-offs involved (P.30, L.549-554).

Response

Regarding Comment 6.1, we have added a new Epidemiological validity subsection to the Study characteristics section (L. 376) to address how researchers justified key parameters like R0 and disease progression rates. Regarding Comment 6.2, this new section also includes a general analytical paragraph and a detailed Calibration and validation methods table summarizing data sources, calibration methods, validation methods, and sensitivity analyses for each study (L. 408-420). This table explicitly confirms the inconsistent calibration rigor across the reviewed studies. Redundant validation information was removed from the Results of the studies table and from texts in the Individual study findings section (L. 421) to avoid duplication. Regarding Comment 6.3, we expanded the modeling discussion in the Summary section (L. 886-901).

Comment 7

A major barrier to the adoption of AI/RL in public health is the "black box" problem. The review briefly mentions "interpretability" in the context of DQN (P.29,L.539-541) but does not sufficiently address this critical issue. The authors should add a dedicated paragraph discussing the interpretability and explainability of the RL policies in the included studies. Were the learned policies analyzed to provide insights (e.g., "lockdown is triggered when hospitalizations exceed X threshold")? How can these models be made trustworthy and actionable for public health officials?

Response

We have added a dedicated Interpretability subsection to the Synthesis of results section (L. 796) to describe how included studies address the “black box” problem and summarize the methods used to make learned policies transparent and actionable for public health officials.

Comment 8

The conclusion calls for prospective validation (P.33, L.630-632). This should be expanded into a discussion on the practical implementation challenges, such as:

(1) Data Latency: Real-world data (cases, hospitalizations) is always delayed. How do these models handle partial and delayed observability?

(2) Behavioral Adaptation: Human behavior changes in response to both the pandemic and the policies themselves. How do these RL models account for this dynamic feedback?

(3) Ethical Considerations: The use of autonomous systems to enforce restrictive policies (like lockdowns) raises significant ethical concerns that should be acknowledged.

Response

Regarding data latency, this challenge is already addressed in the Temporal Delays subsection (L. 660), following the response to Comment 2, and in the corresponding Summary paragraph (L. 858-868), which discusses sequence modeling techniques. Since these sections specifically detail how models handle delayed observability and non-Markovian dynamics, no additional text was added to avoid redundancy. For the points on behavioral adaptation and ethical considerations, a new paragraph has been added to the Summary section (L. 924-939).

Minor Comments

Abstract: The sentence "Across various settings, RL policies outperform heuristic, rule-based, and historical baselines..." (P.3, L.41-43) is a strong claim. Consider qualifying it with "in simulation" or "according to the reviewed studies," to maintain scientific caution.

Table 1: The column "Epidemic Dynamics" could be more precise. For example, "SEIHRD + ABM" should be clarified as "Hybrid (SEIHRD with ABM-governed interactions)" to distinguish it from a pure ABM.

Future Directions: Consider adding counterfactual analysis (using trained RL models to run "what-if" scenarios against historical outbreaks) and integration with ensemble modeling (combining RL with traditional epidemiological ensemble forecasts to improve robustness).

Response

We updated the Abstract with the phrase “according to the reviewed studies” (L. 43). Table 1 (L. 205) was updated to provide more precise descriptions about epidemic modeling, which included recategorizing studies Zong and Luo (2022) and Kompella et al (2020) from Hybrid to pure ABM. This correction was made after a deeper technical review confirmed that their internal SEIR-like structures describe individual agent stochastic transitions rather than population-level compartmental flows. The Epidemic dynamics modeling subsection (L. 251) was updated to reflect this correction. The Conclusions section was expanded to include two mentioned research directions (L. 1002-1006).

Attachment Submitted filename: Response to Reviewers.docx

# Peer review: Decision Letter 1

**DOI:** 10.1371/journal.pone.0351176.r003

**Authors:** Siew Ann Cheong

24 May 2026

Reinforcement learning for policymaking in epidemic control: a scoping review

PONE-D-25-35802R1

Dear Dr. Bolshov,

We’re pleased to inform you that your manuscript has been judged scientifically suitable for publication and will be formally accepted for publication once it meets all outstanding technical requirements.

Within one week, you’ll receive an e-mail detailing the required amendments. When these have been addressed, you’ll receive a formal acceptance letter and your manuscript will be scheduled for publication.

An invoice will be generated when your article is formally accepted. Please note, if your institution has a publishing partnership with PLOS and your article meets the relevant criteria, all or part of your publication costs will be covered. Please make sure your user information is up-to-date by logging into Editorial Manager at Editorial Manager® and clicking the ‘Update My Information' link at the top of the page. For questions related to billing, please contact billing support.

If your institution or institutions have a press office, please notify them about your upcoming paper to help maximize its impact. If they’ll be preparing press materials, please inform our press team as soon as possible -- no later than 48 hours after receiving the formal acceptance. Your manuscript will remain under strict press embargo until 2 pm Eastern Time on the date of publication. For more information, please contact onepress@plos.org.

Kind regards,

Siew Ann Cheong, Ph.D.

Academic Editor

PLOS One

Additional Editor Comments (optional):

Reviewers' comments:

Reviewer's Responses to Questions

**Comments to the Author**

1. If the authors have adequately addressed your comments raised in a previous round of review and you feel that this manuscript is now acceptable for publication, you may indicate that here to bypass the “Comments to the Author” section, enter your conflict of interest statement in the “Confidential to Editor” section, and submit your "Accept" recommendation.

Reviewer #1: All comments have been addressed

**********

2. Is the manuscript technically sound, and do the data support the conclusions?

The manuscript must describe a technically sound piece of scientific research with data that supports the conclusions. Experiments must have been conducted rigorously, with appropriate controls, replication, and sample sizes. The conclusions must be drawn appropriately based on the data presented.

Reviewer #1: Yes

**********

3. Has the statistical analysis been performed appropriately and rigorously?

Reviewer #1: Yes

**********

4. Have the authors made all data underlying the findings in their manuscript fully available?

The PLOS Data policy requires authors to make all data underlying the findings described in their manuscript fully available without restriction, with rare exception (please refer to the Data Availability Statement in the manuscript PDF file). The data should be provided as part of the manuscript or its supporting information, or deposited to a public repository. For example, in addition to summary statistics, the data points behind means, medians and variance measures should be available. If there are restrictions on publicly sharing data—e.g. participant privacy or use of data from a third party—those must be specified.

Reviewer #1: Yes

**********

5. Is the manuscript presented in an intelligible fashion and written in standard English?

PLOS ONE does not copyedit accepted manuscripts, so the language in submitted articles must be clear, correct, and unambiguous. Any typographical or grammatical errors should be corrected at revision, so please note any specific errors here.

Reviewer #1: Yes

**********

6. Review Comments to the Author

Please use the space provided to explain your answers to the questions above. You may also include additional comments for the author, including concerns about dual publication, research ethics, or publication ethics. (Please upload your review as an attachment if it exceeds 20,000 characters)

Reviewer #1: This paper presents a systematic review of deep reinforcement learning (RL) applications for designing non-pharmaceutical interventions (NPIs) in epidemic control. The authors synthesize evidence from peer-reviewed studies (2014–2025) retrieved from major databases, ultimately including 13 eligible studies. The review maps modeling choices, algorithmic architectures, evaluation strategies, and reported outcomes. The findings indicate that most studies employ value-based or policy-gradient methods, often within compartmental or agent-based epidemic simulations. Reward functions commonly balance epidemiological severity with socio-economic costs, and several works incorporate temporal modeling techniques. Across settings, RL-based policies generally outperform heuristic or rule-based baselines in reducing infections, deaths, or lockdown duration while mitigating economic loss. Overall, the paper provides a structured and timely synthesis of RL-based epidemic policy design, while clearly identifying methodological gaps and directions for future research.

The authors have addressed the reviewers’ concerns in the revised manuscript.

**********

7. PLOS authors have the option to publish the peer review history of their article (what does this mean?). If published, this will include your full peer review and any attached files.

If you choose “no”, your identity will remain anonymous but your review may still be made public.

**Do you want your identity to be public for this peer review?** For information about this choice, including consent withdrawal, please see our Privacy Policy.

Reviewer #1: No

**********

# Editor report: Acceptance letter

**DOI:** 10.1371/journal.pone.0351176.r004

**Authors:** Siew Ann Cheong

PONE-D-25-35802R1

PLOS One

Dear Dr. Bolshov,

I'm pleased to inform you that your manuscript has been deemed suitable for publication in PLOS One. Congratulations! Your manuscript is now being handed over to our production team.

At this stage, our production department will prepare your paper for publication. This includes ensuring the following:

* All references, tables, and figures are properly cited

* All relevant supporting information is included in the manuscript submission,

* There are no issues that prevent the paper from being properly typeset

You will receive further instructions from the production team, including instructions on how to review your proof when it is ready. Please keep in mind that we are working through a large volume of accepted articles, so please give us a few days to review your paper and let you know the next and final steps.

Lastly, if your institution or institutions have a press office, please let them know about your upcoming paper now to help maximize its impact. If they'll be preparing press materials, please inform our press team within the next 48 hours. Your manuscript will remain under strict press embargo until 2 pm Eastern Time on the date of publication. For more information, please contact onepress@plos.org.

You will receive an invoice from PLOS for your publication fee after your manuscript has reached the completed accept phase. If you receive an email requesting payment before acceptance or for any other service, this may be a phishing scheme. Learn how to identify phishing emails and protect your accounts at https://explore.plos.org/phishing.

If we can help with anything else, please email us at customercare@plos.org.

Thank you for submitting your work to PLOS ONE and supporting open access.

Kind regards,

PLOS ONE Editorial Office Staff

on behalf of

Dr. Siew Ann Cheong

Academic Editor

PLOS One
